# Catalytic Furfural/5-Hydroxymethyl Furfural Oxidation to Furoic Acid/Furan-2,5-dicarboxylic Acid with H_2_ Production Using Alkaline Water as the Formal Oxidant

**DOI:** 10.1021/jacs.1c10908

**Published:** 2022-01-10

**Authors:** Sayan Kar, Quan-Quan Zhou, Yehoshoa Ben-David, David Milstein

**Affiliations:** Department of Molecular Chemistry and Materials Science, The Weizmann Institute of Science, Rehovot 76100, Israel

## Abstract

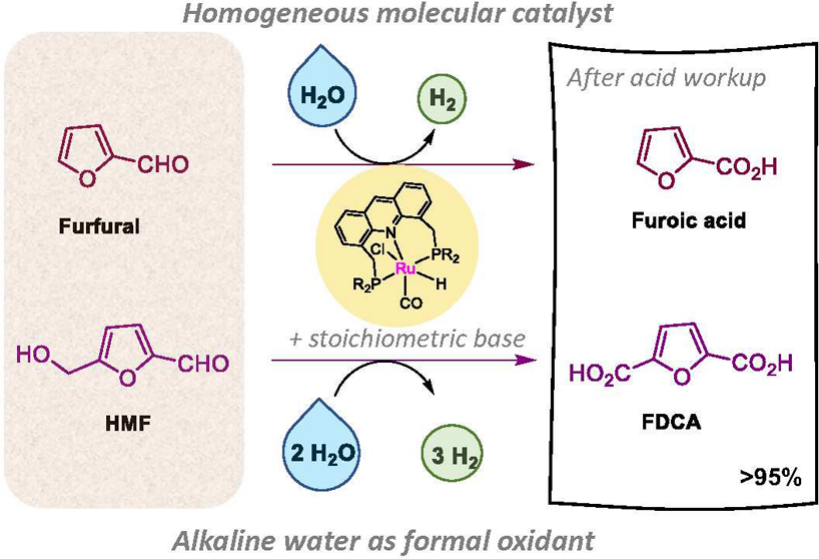

Furfural and 5-hydroxymethyl furfural (HMF) are abundantly available biomass-derived renewable chemical feedstocks, and their oxidation to furoic acid and furan-2,5-dicarboxylic acid (FDCA), respectively, is a research area with huge prospective applications in food, cosmetics, optics, and renewable polymer industries. Water-based oxidation of furfural/HMF is a lucrative approach for simultaneous generation of H_2_ and furoic acid/FDCA. However, this process is currently limited to (photo)electrochemical methods that can be challenging to control, improve, and scale up. Herein, we report well-defined ruthenium pincer catalysts for direct homogeneous oxidation of furfural/HMF to furoic acid/FDCA, using alkaline water as the formal oxidant while producing pure H_2_ as the reaction byproduct. Mechanistic studies indicate that the ruthenium complex not only catalyzes the aqueous oxidation but also actively suppresses background decomposition by facilitating initial Tishchenko coupling of substrates, which is crucial for reaction selectivity. With further improvement, this process can be used in scaled-up facilities for a simultaneous renewable building block and fuel production.

## Introduction

**Figure 1:**
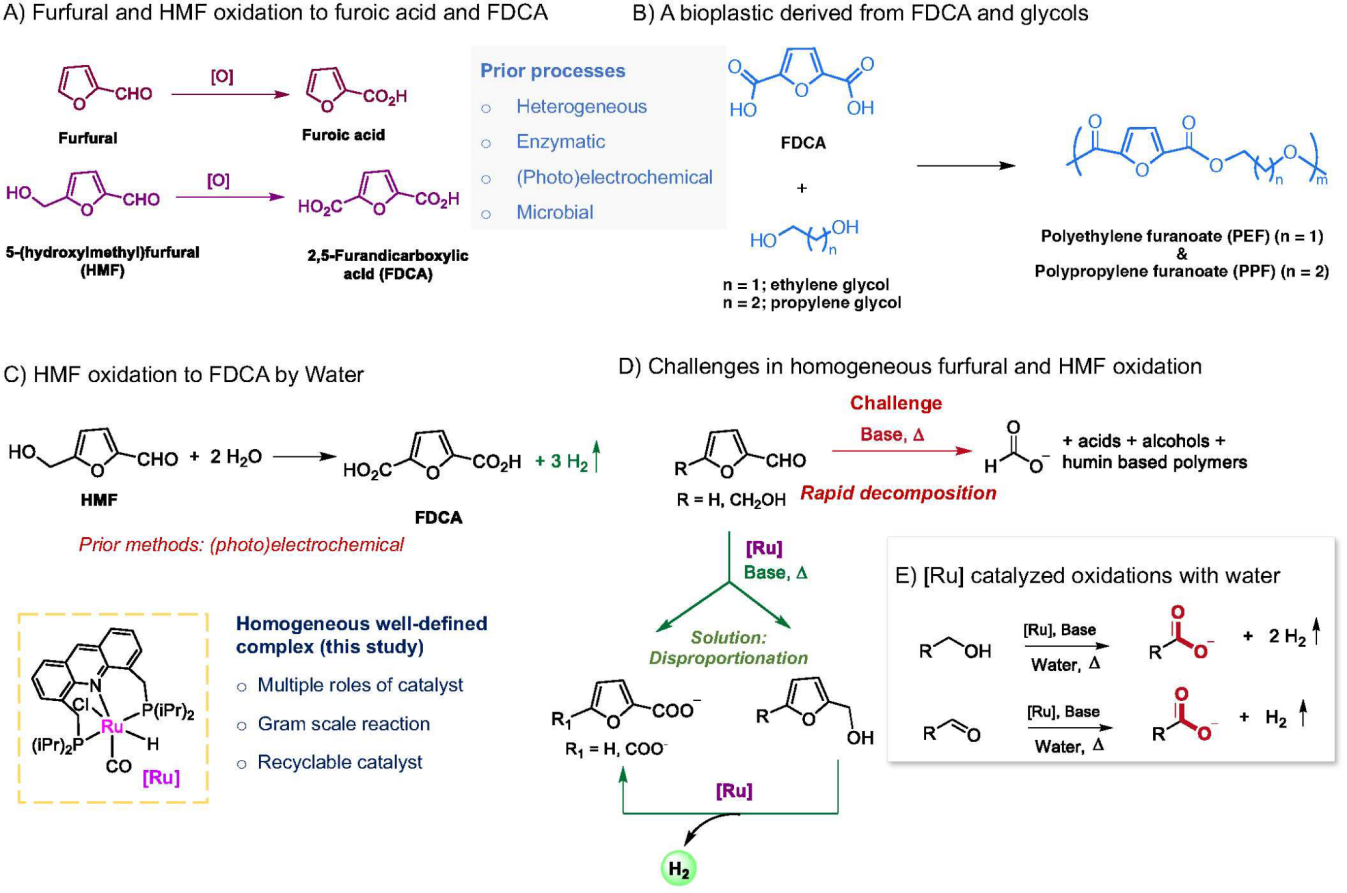
Different aspects of furfural and HMF oxidation to furoic acid and FDCA. (A) Chemical equations with previous approaches. (B) Bioplastics (PEF and PPF) derived from FDCA. (C) HMF oxidation by water to FDCA with H_2_ evolution with previous and new approach. (D) Challenges faced in this study and its circumvention. (E) Relevant ongoing catalytic aqueous oxidation reactions during the process.

Owing to the negative consequences of fossil fuel use, intensive research is ongoing, focusing on transitioning toward a renewable framework for fuel and materials production.^1−5^ Furfural and 5-hydroxymethyl furfural (HMF) are chemical feedstocks produced by hydrolysis of biomass waste.^6−8^ Because of their renewable nature, the synthesis of commodity chemicals from furfural and HMF has garnered increasing attention.^9−12^ Among many products obtainable from furfural and HMF, their oxidation products, furoic acid and furan dicarboxylic acid (FDCA), respectively, hold particular interest (Figure 1A). Furoic acid has many applications including plastic plasticizer, food preservative, pharmaceutical intermediate, and FDCA precursor, and has potential applications in optics technology because of its unique crystal properties, with large-scale synthesis plants operated by multiple companies.^13−16^ Similarly, FDCA is a promising renewable alternative to terephthalic acid for polymers synthesis.^17^ FDCA-based renewable biopolymers often show improved mechanical, thermal, and gas transport properties compared to their terephthalic acid based counterparts found in the market (Figure 1B),^18,19^ with the United States Department of Energy identifying FDCA as 1 of the 12 priority chemicals for the establishment of a green chemical industry in the future.^20,21^ Several companies started pilot plants for FDCA synthesis from HMF over the past decade because of its growing market in the polymer industry; however, the markedly different approaches undertaken reflect the lack of an economically optimized process for the desired synthesis.^22^

The most explored selective oxidative routes to access furoic acid and FDCA from furfural and HMF use heterogeneous catalysts, such as supported PbPt/C, Au, Ag_2_O/CuO, and AuPd/Mg(OH)_2_, with excess oxidants (mainly high-pressure oxygen or air).^23−26^ The process produces water as the side product for HMF to FDCA conversion, which although environmentally benign, does not hold any economic value. Alternative oxidation methods such as electrochemical^27−29^ and bio^30^ -enzymatic^31−34^ oxidation of furfural and HMF to furoic acid and FDCA have also been explored. Recent reports have elegantly coupled H_2_ production from water with biomass oxidation to (photo)electrochemically produce H_2_ and furoic acid/FDCA from water and furfural/HMF mixture (Figure 1C).^35−40^ These photoelectrochemical systems, however, require advanced specialized materials and can be challenging to rationally improve. Besides, their large-scale implementation can be difficult because of the need for sophisticated infrastructures and low working concentrations.^41^

In contrast to the explored heterogeneous, biological, enzymatic, and (photo)electrochemical processes, catalytic homogeneous systems for furfural and HMF oxidation are extremely limited. The use of well-defined homogeneous complexes for furfural/HMF oxidation is challenging because of the facile substrate decomposition pathways at high temperatures in alkaline/aerobic conditions leading to the formation of polymeric products (Figure 1D).^42,43^ Goldberg and co-workers have reported complexes that are active in catalyzing the aqueous reforming of other aldehydes to acids, but display minimal activities when furfural/HMF is used as a substrate.^44,45^ Interestingly, Nakajima and co-workers have recently reported an N-heterocyclic carbene organocatalyst for furfural to furoic acid conversion in the presence of 1,8-diazabicyclo[5.4.0]undec-7-ene (DBU) base, which use O_2_ as the oxidant, but can only partially oxidize HMF to 5-hydroxymethylfurancarboxylic acid intermediate.^46^

Herein, we report the catalytic homogeneous oxidation of furfural and HMF to furoic acid and FDCA, respectively, using alkaline water as the formal oxidant. The reaction is catalyzed by well-defined ruthenium complexes with acridine-based PNP pincer ligands^47,48^ and generates pure H_2_ gas as the reaction byproduct. Mechanistic studies indicate that the Ru complexes not only catalyze the substrate oxidation to acid but also induce rapid substrate disproportionation in the initial hour, which is crucial in preventing substrate decomposition. Further reaction involves the dehydrogenative oxidation of the generated alcohol by water (Figure 1E). The scalability of the process is demonstrated by carrying out a gram-scale reaction. Notably, the system can theoretically produce up to a substantial 3.48 wt % H_2_ when HMF is used as a substrate and LiOH as the base if a neat system is developed, generating both renewable fuel and material precursors in one simple homogeneous process.

## Results and Discussion

### Furfural Oxidation to Furoic Acid

#### Catalyst Screening

**Figure 2:**
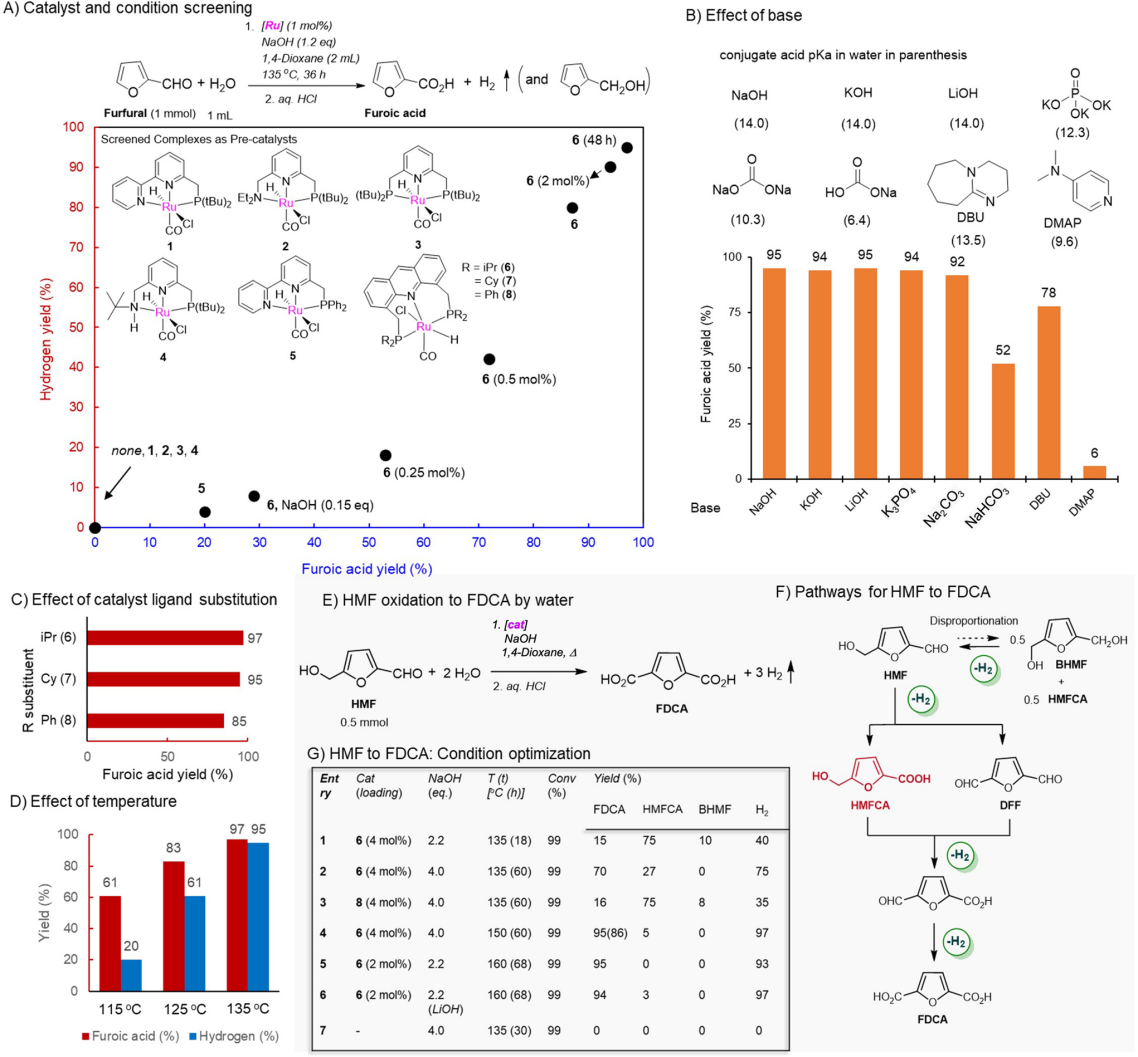
Catalytic oxidation of furfural and HMF with water as the formal oxidant. (A–D) Furfural to furoic acid: (A) Catalyst and condition screening. (B) Effect of base. (C) Catalyst ligand substitution effect. (D) Temperature effect on furfural to furoic acid conversion. (E–G) HMF to FDCA: (E) Reaction equation. (F) Different possible parallel pathways. (G) Condition optimization to obtain FDCA and H_2_. Reactions in (A)–(D) were conducted using 1 mmol of furfural, 1 mol % catalyst, and 1.2 equiv of the base in 1,4-dioxane (2 mL)/water (1 mL) unless otherwise specified. Reactions in (B) and (D) are with **6** (1 mol %) as the catalyst, and reactions in (C) used NaOH (1.2 equiv) as the base with 48 h reaction time. Reactions in (G) used 0.5 mmol of HMF in 1,4-dioxane (2 mL)/water (1 mL), with other reaction conditions as specified. Yields calculated by ^1^H NMR (mesitylene standard) and gas buret (H_2_). Yields correspond to furoic acid or FDCA salts before acidification with isolated yields in parentheses.

Our investigation started by exploring the aqueous furfural oxidation to furoic acid in the presence of the Ru-PNN bipyridyl complex **1** (Figure 2A), which is reported by us to catalyze aqueous dehydrogenative oxidation of alcohols to carboxylic acid salts by alkaline water (Table S1, Supporting Information).^49^ However, attempts toward aqueous oxidation of furfural at 135 °C by complex **1** (1 mol %) in 1,4-dioxane/alkaline water resulted in complete decomposition of furfural with no generation of H_2_ or furoic acid. A control reaction revealed that furfural is prone to degradation at elevated temperature under the reaction conditions, even without any catalyst. We subsequently screened several ruthenium-based pincer complexes developed in our group for the desired dehydrogenative oxidation reaction. However, all efforts involving complexes **1–4** resulted in substrate decomposition with no significant gas generation (Figure 2A). In the case of the Ru-PNNBPy^Ph^ complex (**5**), the furfural decomposition rate slowed down, obtaining the acid and alcohol as the reaction products, however, with only a small amount of H_2_ generated (8%) (Table S1, entry 7). Remarkably, the acridine PNP complex **6** catalyzed the reaction with high H_2_ (80%) yield and furoic acid (87%) yield (Table S1, entry 8). GC analysis of the generated gas mixture showed only H_2_ gas with no CO contamination, suitable for its use in a proton-exchange membrane (PEM) fuel cell without further purification. Optimization of the catalyst amount showed that 1 mol % catalyst loading is ideal for both high H_2_ and furoic acid yields, with lower catalyst loadings being detrimental, especially for H_2_ yields (Figure 2A). A stoichiometric base was necessary for the reaction, and under the catalytic base, decreased yields were observed. Furoic acid and H_2_ were obtained in >95% yield by increasing the reaction time from 36 to 48 h.

#### Effect of the Base, Catalyst Substitution, and Temperature

Next, the effect of different bases was explored (Figure 2B; Table S2). Strong bases such as NaOH, KOH, and LiOH were effective for high furoic acid and H_2_ yield, with relatively weaker bases such as K_3_PO_4_ and Na_2_CO_3_ being similarly effective (>90% furoic acid yield). Decreasing the base strength further as with NaHCO_3_ resulted in decreased furoic acid yield (52%), with the rest of the product being the alcohol. Among amine-based organic bases, DBU was moderately effective for acid generation (78%), whereas with dimethyl aminopyridine (DMAP), almost no acid formed, with 85% furfural being unreacted. We also explored the effect of different ligand substituents of the catalyst structure on product yield (Figure 2C). Complexes Ru-Acr(^i^Pr) (**6**) and Ru-Acr(Cy) (**7**) displayed similar catalytic activities under the conditions for furfural oxidation to furoic acid. In contrast, the Ru-Acr(Ph) complex **8**, with electron-withdrawing Ph substitutions onto phosphorus donor atoms, was slightly less active in catalyzing the reaction. Optimization studies regarding reaction temperature revealed that 135 °C is required for reaction completion in 48 h and decreasing the temperature to 125 or 115 °C resulted in lower yields (Figure 2D).

### HMF Oxidation to FDCA

The direct oxidation of HMF to FDCA via this homogeneous dehydrogenative aqueous oxidation method was subsequently explored (Figure 2E). As mentioned earlier, direct FDCA synthesis from HMF by water is a process with a great prospect in the industrial production of biobased renewable polymers and fuels, for which current methods are limited. HMF oxidation to FDCA can occur via two different routes— one via the generation of 5-hydroxymethyl-2-furancarboxylic acid (HMFCA) from the initial oxidation of the aldehyde group to acid or via initial oxidation of the alcohol group in HMF forming diformylfuran (DFF), whose subsequent oxidation generates FDCA (Figure 2F). With use of our method, heating HMF at 135 °C in the presence of complex **6** (4 mol %), NaOH (2.2 equiv) in 1,4-dioxane/water (2:1 mL/mL), FDCA formation in 15% yield was observed after 18 h (Figure 2G, entry 1). The primary reaction product was the HMFCA intermediate (75%), signifying that oxidation of the aldehyde group is easier than that of the alcohol group under the conditions (Figure S12). These two products, along with the disproportionation product bis(hydroxymethyl)furan (BHMF, 10%), accounted for all the HMF conversion (99%), indicating the absence of any polymeric side pathways. A higher yield of FDCA (70%) was obtained by using a more alkaline solution (4 equiv of base) and a longer reaction time (60 h) (entry 2). Similar to furfural oxidation, complex **8** was less active for HMF to FDCA oxidation, too (entry 3). FDCA yield increased to 95% when the reaction temperature was increased to 150 °C, using **6** as the catalyst (entry 4). Under optimized conditions, FDCA in high yield (95%) was obtained (H_2_ yield: 93%) with 2 mol % of complex **6** and 2.2 equiv of NaOH, at 160 °C after 68 h of reaction (entry 5). LiOH was similarly active as NaOH as a base in facilitating FDCA formation under the reaction conditions (entry 6). Thus, it is shown that complex **6** can catalyze the direct and selective HMF oxidation to FDCA in the presence of alkaline water with high yields while also generating quantitative pure H_2_ gas. In the absence of catalyst, decomposition of HMF into unidentifiable products was observed (entry 7).

### Mechanistic Investigation

**Figure 3:**
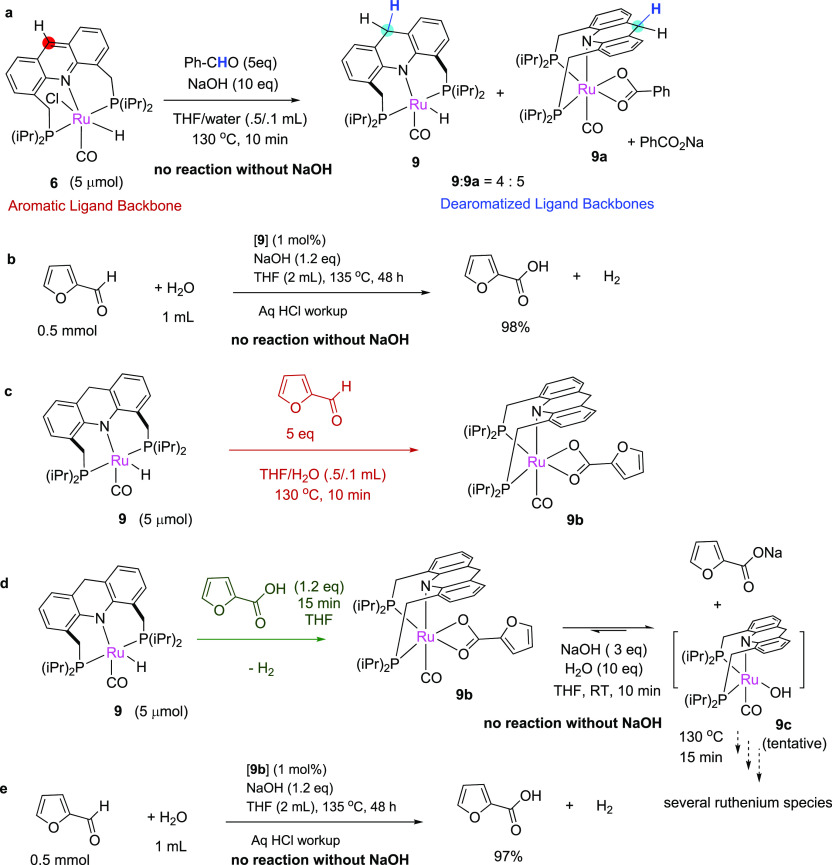
Mechanistic and control experiments. (a) Reactivity of complex **6** with benzaldehyde in the presence or absence of NaOH. (b) Complex **9** catalyzed furfural oxidation to furoic acid. (c) Reactivity of complex **9** with furfural and water. (d) Reactivity of **9** with furoic acid to generate the furoate complex **9b**. Additional reactivity of **9b** in the presence of base NaOH and water. No reaction took place without the base. (e) Catalytic activity of complex **9b** in catalyzing furfural to furoic acid in the presence of NaOH.

We subsequently explored the reactivity complex **6** with aldehyde, base, and water to understand the reaction mechanism (Figure 3). Initial experiments were carried out with benzaldehyde as the furfural surrogate, which is more stable at higher reaction temperatures and easier to follow. Complex **6** does not react with benzaldehyde (5 equiv) under neutral conditions in a THF/water solvent mixture (0.5:0.1 mL/mL), even when heated at a high temperature of 130 °C for 0.5 h (Figure 3a). On the other hand, when NaOH (5 equiv) was added, and the solution was subsequently heated at 130 °C for 10 min inside a J. Young NMR tube, generation of two new complexes were observed in the ^31^P{^1^H} NMR spectrum (Figure S31) with their characteristics ^31^P chemical shifts at 74.1 and 87.5 ppm, respectively, at a 0.8:1.0 ratio (parent complex ^31^P signal chemical shift is at 69.1 ppm). In the ^1^H NMR spectrum, surprisingly, the 9H acridine aromatic protons from both complexes were missing, which appear around 8–9 ppm as a singlet, with new sets of peaks around 3.5–4 ppm, which were assigned to CH_2_ protons at the 9 position of the acridine ring (Figure S33). On the basis of further NMR analysis and our previously reported observations with the Ru-Acr system, these two complexes were identified as the dearomatized 9H acridine complex (**9**) and Ru-Acr9H phenyl-carboxylate complex (**9a**) (Figure 3a).^47^ Thus, under the reaction conditions, hydride transfer from the substrate initially takes place to the 9CH position of the catalyst’s acridine backbone, leading to the formation of dearomatized complexes,^50,51^ which further catalyze the reaction.

Accordingly, complex **9** catalyzed the dehydrogenative oxidation of furfural under basic conditions with similar activity as the aromatized complex **6** (furoic acid yield 98%, Figure 3b). Subsequent mechanistic experiments focused on the reactivity of complex **9** with different substrates. In the absence of base, complex **9** reacts with water and furfural at 130 °C to generate the furoate complex **9b** after 10 min (Figure 3c, Figure S35). **9b** can also be accessed alternatively by mixing **9** with furoic acid in THF at room temperature (Figure 3d, Figure S36).^52^**9b** was found to be stable under neutral conditions in THF in the presence of water (10 equiv), even when subjected to high temperature (130 °C) (Figure S37). On the other hand, when NaOH (3 equiv) was added to the solution, the formation of a new complex was observed at RT in the ^31^P NMR, along with the generation of sodium furoate (observed in ^1^H NMR) (Figure 3d). The new complex slowly decomposed at RT, which was facilitated at elevated temperature (Figure S38) and is tentatively assigned the structure of high-energy hydroxide intermediate **9c**. The furoate complex **9b** was observed to catalyze the aqueous oxidation of furfural in the presence of an external base; however, its catalytic activity subsided when no base was present in the system (Figure 3e). Thus, complex **9b** seemingly acts as a deactivating species under the reaction conditions, and the addition of a stoichiometric base is required to remove the chelating furoate ligand to generate the product while at the same time opening relevant coordination sites for catalytic turnover.

**Figure 4:**
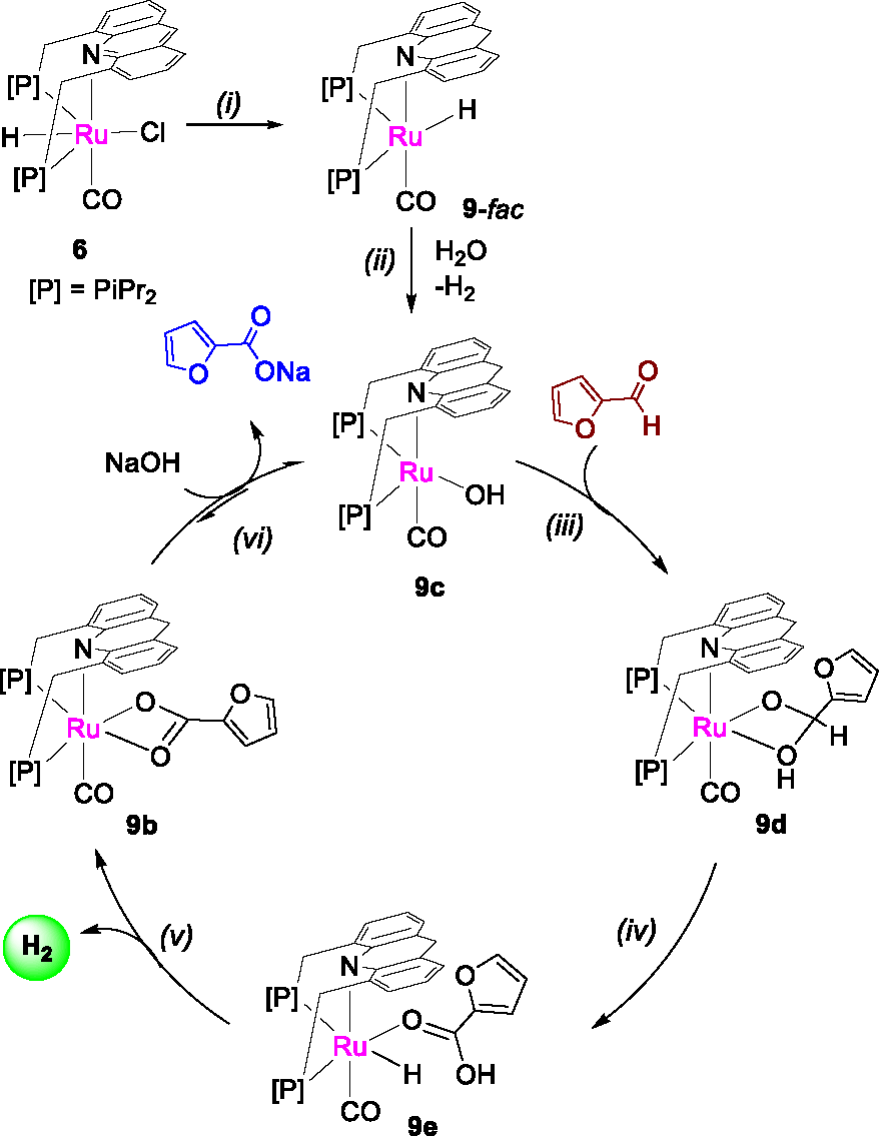
Mechanistic cycle. A plausible scheme for furfural to furoic acid formation using water catalyzed by **6** involving the generation of dearomatized complexes. Elemental steps: (i) initial dearomatization of the catalyst, (ii) initial dehydrogenation of water by **9** generating hydroxide complex, (iii) hydroxide attack on the aldehyde, (iv) beta hydride elimination, (v) H_2_ evolution, and (vi) product elimination and substitution.

On the basis of these observations, a mechanism cycle as shown in Figure 4 is proposed. Initial hydride transfer from the substrate to the catalyst ligand backbone generates the dearomatized complex **9** (step i). Complex **9**, in the presence of water, generates the hydroxide complex **9c** with H_2_ evolution (step ii).^50^ In the presence of furfural, the hydroxo complex generates the hemiacetal complex **9d** via attack of the hydroxide ligand onto furfural (step iii), akin to the mechanism proposed at heterogeneous metal water interfaces.^53−55^ Further β-hydride elimination generates the furoic acid complex (step iv), H_2_ liberation leading to the furoate complex **9b** (step v). In the absence of an external base, the catalytic cycle halts at this stage; however, when a base is present, the furoate ligand detaches as product furoate salt while regenerating the hydroxide complex (step vi). It should be noted here that an alternate mechanism involving the initial free acetal formation, followed by acetal dehydrogenation and beta hydride elimination cannot be entirely ruled out, based on our mechanistic observations (SI, section 7.5).

**Figure 5:**
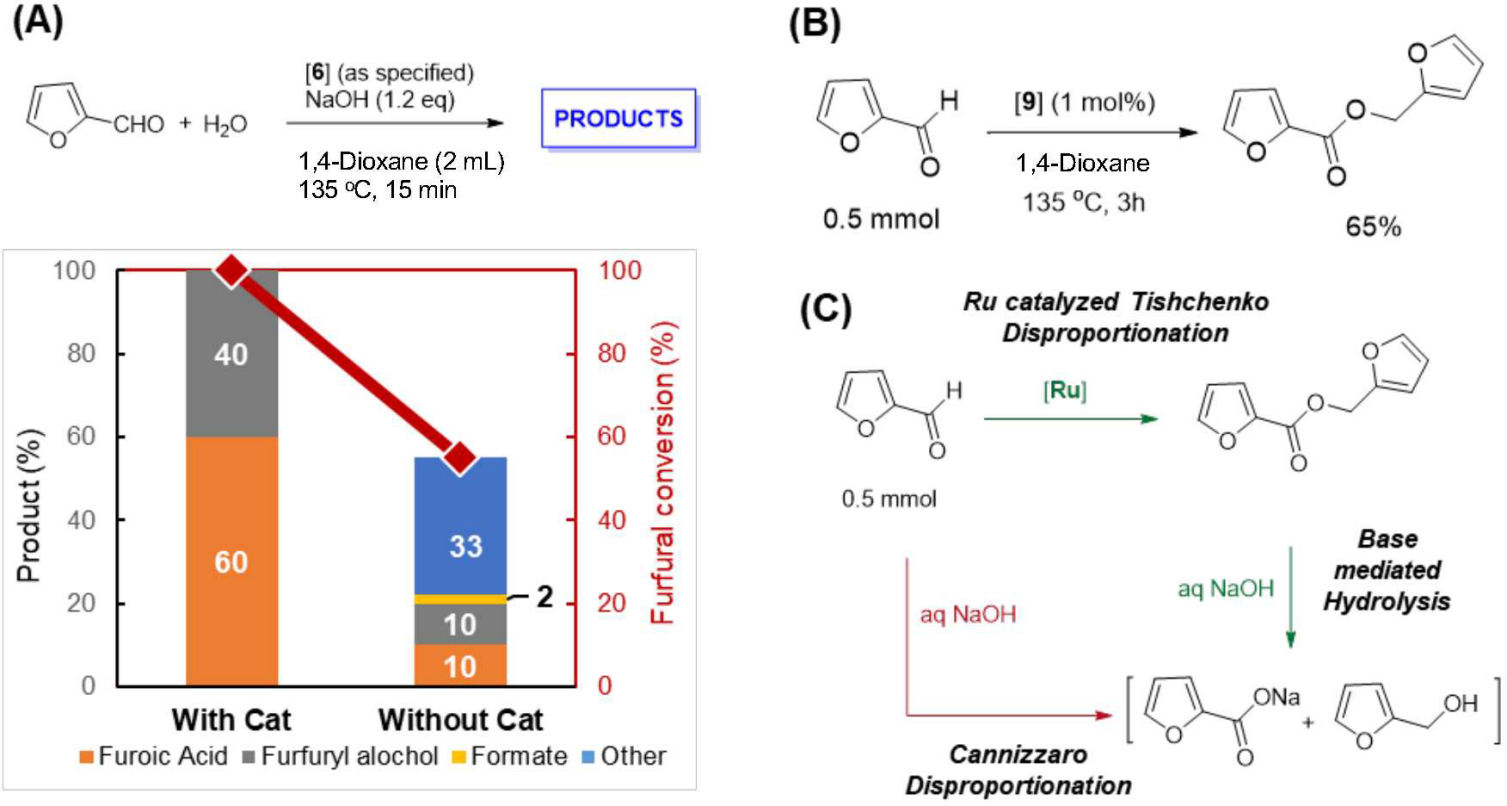
Active decomposition suppression by Ru complexes. (a) Product distribution from furfural oxidation after 15 min with and without the presence of catalyst. Furfural (0.5 mmol), water (1 mL), **6** (1 mol %), NaOH (1.2 equiv), and 1,4-dioxane (2 mL). (b) Catalytic formation of furfuryl furoate by Tishchenko coupling of furfural catalyzed by **9**. Furfural (0.5 mmol), **9** (1 mol %), and 1,4-dioxane (2 mL). (c) Alternative [Ru] and base-mediated pathway for furfural disproportionation under the reaction conditions as compared to Cannizzaro disproportionation.

Simultaneous to the aldehyde dehydrogenative coupling with water, leading to generation of the furoate salt and H_2_, disproportionation of furfural also takes place to generate furoate and furfuryl alcohol as the reaction products. These two processes result in the quick consumption of the initial aldehyde during the reaction. Total consumption of the aldehyde was observed after the initial 15 min of reaction along with 60% of furoic acid and 20% H_2_ yield (40% of furfuryl alcohol side product; Figure 5A). The subsequent reaction completion involves the conversion of the generated furfuryl alcohol to furoic acid. The observed H_2_ evolution time profile suggests that the aldehyde dehydrogenative coupling reaction with water is quick, with alcohol dehydrogenation being comparatively slower (Figure S16). Similar to furfural, HMF oxidation is also surmised to proceed involving a combination of direct dehydrogenative oxidation to FDCA and disproportionation–oxidation pathway involving BHMF. Accordingly, when BHMF was tried as a substrate instead of 5-HMF, FDCA in high yields (81%) was isolated (similar reaction conditions as in Figure 2G, entry 4) (see Supporting Information). The reaction pathways ongoing during the reactions of furfural and HMF oxidation are detailed in Figure S45.

### Active Decomposition Inhibition by [Ru]

The ruthenium complex takes an active part in the substrate disproportionation process. As mentioned, under standard reactions with complex **6**, furfural is entirely consumed in 15 min via combined dehydrogenative aqueous oxidation (to acid and H_2_) and disproportionation (to acid and alcohol) to produce furoate and furfuryl alcohol (Figure 5A), selectively. Interestingly, when the reaction was conducted without catalyst, only 55% furfural conversion was observed after 15 min, with only 20% accounting for acid and alcohol products by Cannizzaro disproportionation, the rest being unidentified decomposition products (Figure 5A). These results suggest a parallel disproportionation pathway for furfural, in addition to the Cannizzaro mechanism, in the presence of Ru catalyst. Accordingly, when furfural was heated at 135 °C in 1,4-dioxane in the presence of complex **9** (1 mol %) for 3 h, formation of furfuryl furoate in 65% yield was observed, signifying that complex **9** can catalyze the Tishchenko coupling of furfural (Figure 5B).^51,56^ The resulting ester, under the reaction conditions of Table S1 is hydrolyzed to furoate salt and furfuryl alcohol (Figure 5C). This alternative disproportionation pathway via Tishchenko coupling followed by base-mediated hydrolysis leads to quick consumption of all furfural/HMF before the onset of decomposition and is crucial for the observed high oxidation selectivity with catalyst **6** or **9**. A tentative mechanism of the Tishchenko reaction catalyzed by complex **9** is shown in Supporting Information (Figure S40).

### Catalyst Recycling and Scale-Up

Focusing on the practicality of the system for large-scale implementation, we also explored the possibility of catalyst recycling after the reaction. The catalyst was recovered from the postreaction solution by evaporation of the solvents and extracting the catalyst with benzene (detailed procedure in Supporting Information). Following this protocol, the catalyst was recycled for three cycles and its catalytic activities for furfural oxidation were retained after the third cycle. (97%, 91%, and 83% sodium furoate yield, respectively, in the first, second, and third cycle), demonstrating the viability of catalyst recycling. We also conducted a gram-scale experiment with 15 mmol of furfural (1.44 g) to check the scalability of the process. After 68 h of reaction at 150 °C, 1.3 g of furoic acid (77% yield) was isolated from the reaction, along with 316 mL of H_2_ collected (87% yield), demonstrating the scalability of the process.

## Conclusions

We report here molecular catalysts for the direct catalytic oxidation of furfural and HMF to furoic acid and FDCA, respectively, using alkaline water as the formal oxidant. The oxidation is associated with the generation of pure H_2_ gas with no detectable CO contamination (detection limit: 15 ppm), suitable for direct utilization in a proton-exchange membrane fuel cell. When the ruthenium acridine PNP complex **6** was used as the catalyst, furoic acid/FDCA was obtained with high yield (>95%) by aqueous oxidation of furfural/HMF. Mechanistic studies revealed an initial hydride transfer from the substrate to the catalyst ligand backbone under the conditions, generating the dearomatized complex **9**, which subsequently catalyzed the oxidation. Complex **9** also catalyzes the Tishchenko coupling of substrates, which is essential for background decomposition suppression. Overall, the Ru-acridine PNP-based system is unique, with its atypical reactivity, in catalyzing the selective furfural/HMF oxidative reactions with complete inhibition of substrate decomposition, resulting in high furoic acid/FDCA and H_2_ yields. We believe that this report will initiate further investigations toward the homogeneous catalytic oxidation of furfural/HMF, largely overlooked until now, especially with water as the formal oxidant, given the dire importance of transitioning toward renewable material and fuel synthesis in the context of modern sustainability. With sufficient improvements in the conditions, such a homogeneous process could be ideal for large-scale FDCA (and furoic acid) and H_2_ synthesis from the HMF (and furfural)–water mixture in an industrial setup, compared to the equivalent (photo)electrochemical processes.

## Supporting Information Available

Materials and methods, synthesis and characterization data, experimental procedures, NMR spectra, mechanistic experiments, and general discussion (PDF)
