# Supplementary material for: Catalytic Furfural/5-Hydroxymethyl Furfural Oxidation to Furoic Acid/Furan-2,5-dicarboxylic Acid with H2 Production Using Alkaline Water as the Formal Oxidant
Source: J Am Chem Soc. 2022 Jan 10;144(3):1288–95. doi: 10.1021/jacs.1c10908 (PMC8796234; doi:10.1021/jacs.1c10908)
Supplement: Supplementary file 1 — ja1c10908_si_001.pdf [file ja1c10908_si_001.pdf]

Catalytic Furfural/5-Hydroxymethyl Furfural Oxidation to Furoic acid/Furan-2,5-dicarboxylic Acid with H_2 Production Using Alkaline Water as the Formal Oxidant

Sayan Kar,[#] Quan-Quan Zhou,[#] Yehoshoa Ben-David, and David Milstein*

Department of Molecular Chemistry and Materials Science, The Weizmann Institute of Science, Rehovot 76100, Israel.

[#]These authors contributed equally.

Correspondence to: david.milstein@weizmann.ac.il

Table of contents

1. Materials and methods 2
2. Synthesis and characterization of complex **8** 3
3. Standard procedure for furfural oxidation to furoic acid using water 10
4. Standard procedure for HMF oxidation to FDCA using water 16
5. Procedures for additional experiments 19
6. Mechanistic experiments 27
7. General discussion 40
8. References 47

1. Material and methods

All experiments were carried out under an inert atmosphere (with N_2 or Ar) using standard Schlenk techniques. Complexes RuHCl(CO)$P^{tBu}NN_{Bpy}$ (**1**),[1] RuHCl(CO)$P^{tBu}NN_{Et}$ (**2**),[2] RuHCl(CO)PNP^{tBu} (**3**),[3] RuHCl(CO)$P^{tBu}NN_{tBu}$H (**4**),[4] RuHCl(CO)$P^{Ph}NN^{Bpy}$ (**5**),[5] RuHCl(CO)AcrPNP^{iPr} (**6**),[6] RuHCl(CO)AcrPNP^{Cy} (**7**),[7] and RuHCl(CO)dAcrPNP^{iPr} (**9**)[8] were prepared according to the previously reported procedures. Synthesis procedure of complex RuHCl(CO)AcrPNP^{Ph} (**8**) is given in the subsequent section. All catalysts were weighed inside nitrogen filled glove box. Furfural was purchased from Sigma Aldrich and passed through a silica column to remove brown impurities and stored inside N_2 glove box. 5-Hydroxylmethylfurfural (HMF) was purchased from S.L. Moran and used without further purification. Reagent grade sodium hydroxide (NaOH), potassium hydroxide (KOH), lithium hydroxide (LiOH), sodium carbonate (Na_2CO_3), sodium bicarbonate ($NaHCO_3$), potassium phosphate (K_3PO_4), 1,8-Diazabicyclo[5.4.0]undec-7-ene (DBU), dimethyl aminopyridine (DMAP) were purchased from commercial sources and used without further purification. All solvents were purified according to standard procedures under an argon atmosphere, bubbled with argon, and stored over 4 Å molecular sieves (MS). Furfural and DBU were bubbled with argon for half an hour prior to their use. 1,3,5-trimethylbenzene (mesitylene) was purchased from commercial sources and used as received. Deionized water was used in the reaction, which was bubbled with argon for half an hour prior to its use.

NMR spectra were recorded at room temperature on a Bruker AMX-300 (300 MHz) or AMX-400 (400 MHz) or AMX-500 (500 MHz) spectrometers. Chemical shifts of the NMR spectra are reported relative to residual signals of $CDCl_3$ (^{1}H NMR: $\delta = 7.26$ ppm, ^{13}C NMR: $\delta = 77.16$ ppm), benzene-D_6 (^{1}H NMR: $\delta = 7.16$ ppm, ^{13}C NMR: $\delta = 128.06$ ppm), or DMSO-d_6 (^{1}H NMR: $\delta = 2.5$ ppm, ^{13}C NMR: $\delta = 40.00$ ppm) or the internal standard mesitylene. $^{31}P\{^1H\}$ NMR chemical shifts are reported in ppm downfield from H_3PO_4 and referenced to an external 85% solution of phosphoric acid in D_2O. GC–MS was carried out on HP 6890 (flame ionization detector and thermal conductivity detector) and HP 5973 (MS detector) instruments equipped with a 30 m column (Restek 5MS, 0.32 mm internal diameter) with a 5% phenylmethylsilicone coating (0.25 mm) and helium as carrier gas. IR spectra were recorded on a Nicolet FTIR spectrophotometer (KBr, thin Film). GC was carried out on HP 6890 or Agilent 7890B Series GC System with N_2 or Helium as carrier gas.

2. Synthesis and characterization of complex 8

2.1. Synthesis of the ligand

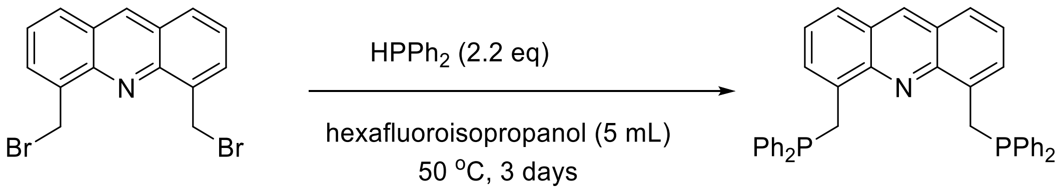

Procedure. Inside a N_2 glove box, 1 mmol (365 mg) of acridine dimethylenebromide was dissolved in 5 mL of hexafluoro-2-propanol, to which 2.2 mmol (410 mg) of diphenylphosphine was added dropwise. The solution was transferred to a pressure tube and was heated at 50 °C for 3 days. Afterwards, it was cooled down to room temperature, and 20 mL DCM (dichloromethane) was added. The solution was *quickly** washed with 30 mL of 10% aqueous NaOH. The organic layer was collected, and the aqueous layer was washed once more with 20 mL DCM. The combined DCM solutions were dried with $MgSO_4$, and DCM was removed, affording a crude yellow solid.

The solid was then taken inside glove box and dissolved in 5 mL THF. The solution was filtered through a small Celite pad. THF was then removed, and the resulting yellow solid was washed with pentane, sparing amounts of ether, and methanol to obtain the pure PNP(Ph) acridine ligand in ~70% yield.

**NOTE: If not done quickly, the product ligand can by oxidize under atmospheric oxygen, forming the corresponding phosphine oxide. The phosphine oxide can be removed at a later stage by washing with excess methanol, however, it also results in significant decrease in yield of the desired ligand.*

^{31}P NMR (162 MHz, Chloroform-*d*) δ -9.69.

^{1}H NMR (400 MHz, Chloroform-*d*) δ 8.76 (s, 1H), 7.89 (d, J = 8.2 Hz, 2H), 7.52 (t, 8H), 7.43 – 7.23 (m, 16H), 4.42 (d, J = 1.7 Hz, 4H).

$^{13}C\{^{1}H\}$ NMR (101 MHz, Chloroform-*d*) δ 146.60, 139.34 (d, J = 16.7 Hz), 136.68 (d, J = 6.2 Hz), 136.14, 133.06 (d, J = 18.7 Hz), 129.72 (d, J = 9.3 Hz), 128.37, 128.24 (d, J = 6.4 Hz), 126.63, 126.23, 125.26, 31.20 (d, J = 15.2 Hz).

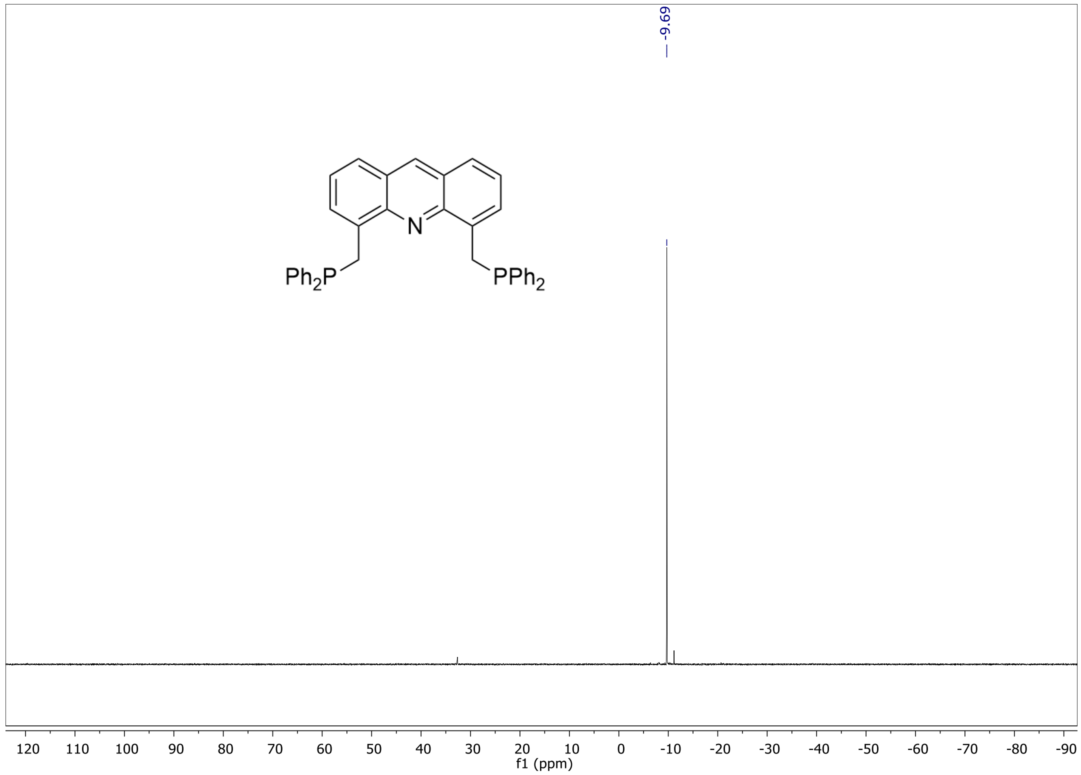

Figure S1. $^{31}P\{^{1}H\}$ NMR spectrum (162 MHz) of PNP(Ph) acridine ligand in $CDCl_3$.

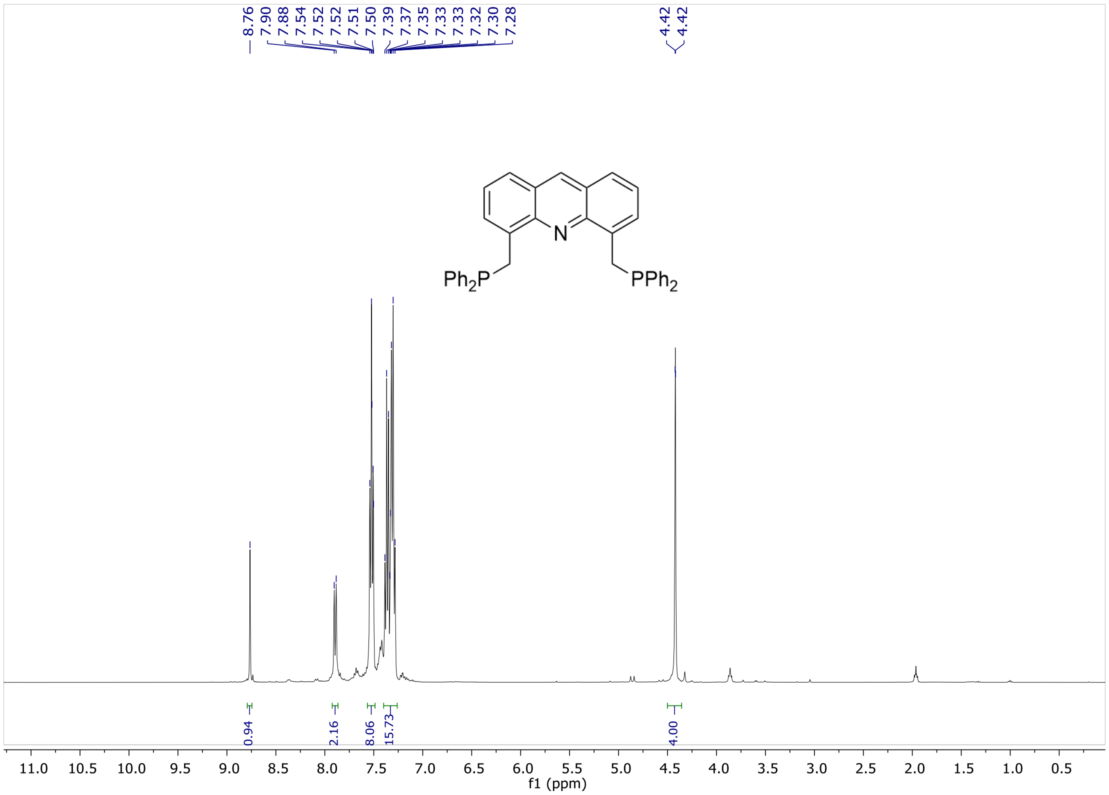

Figure S2. ^{1}H NMR spectrum (400 MHz) of PNP(Ph) acridine ligand in $CDCl_3$.

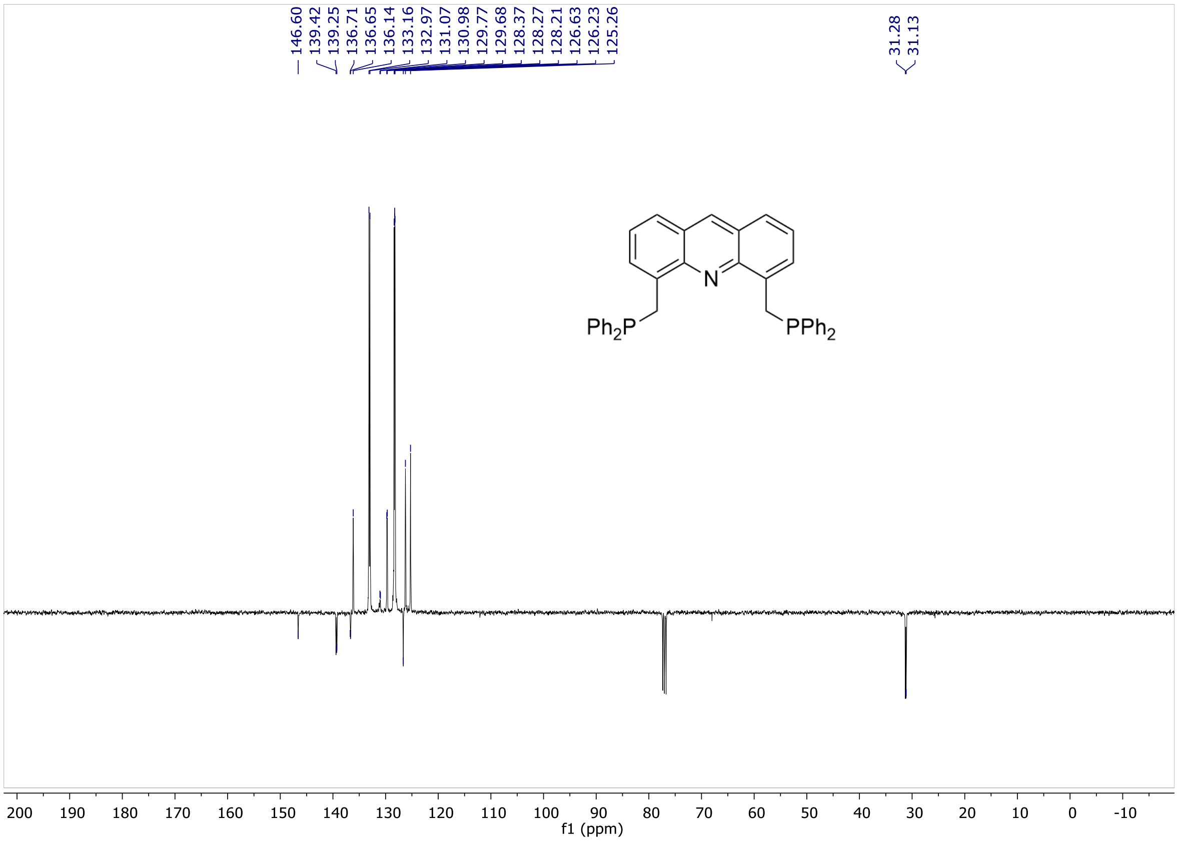

Figure S3. DEPT 135 NMR spectrum (101 MHz) of PNP(Ph) acridine ligand in $CDCl_3$.

__2.2. Synthesis of the acridine complex__ RuH(CO)ClAcrPNP(Ph) (**8**)

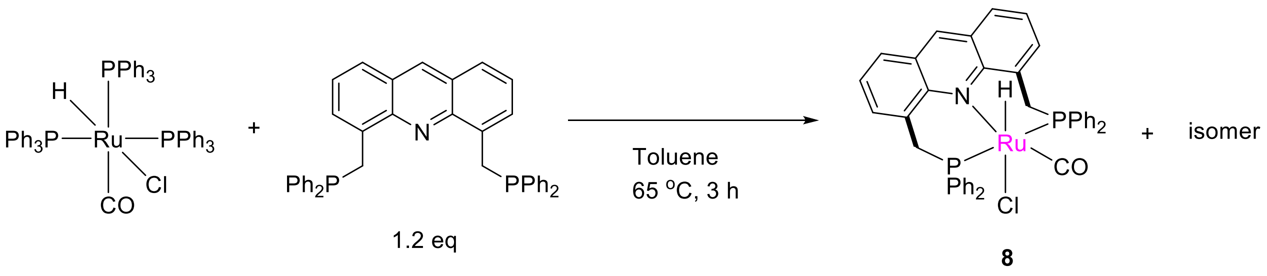

Procedure: Inside a N_2 glove box, 0.1 mmol of $RuHCl(CO)(PPh_3)_3$ (95.2 mg) and 0.12 mmol of the PNP(Ph) acridine ligand (69 mg) were suspended in 5 mL toluene. The solution was transferred to a pressure tube and was heated at 65 °C for 3 hours. Formation of yellow precipitates were observed during the reaction. After 3 hours, the solution was cooled to room temperature and toluene was evaporated *in vacuo*. The resulting yellow solid was washed with diethyl ether and THF for multiple times, affording complex **8** as a yellow powder in 89% yield as mixtures of possible *fac* and *mer* isomers.

The complex is not soluble in less polar solvents such as benzene, toluene, or THF. NMR analysis of a CD_2Cl_2 solution showed the presence of two major isomers, along with one minor isomer, which was directly used for catalysis. The possibility of different isomers with the ruthenium acridine PNP^{iPr} framework has been discussed before in the context of computational studies.[9] It is thus likely that while for the acridine PNP^{iPr} complex only one isomer is thermodynamically favorable, for the PNP^{Ph} complex, their energies are much more similar, leading to their co-observations.

$^{31}P\{^{1}H\}$ NMR (162 MHz, CD_2Cl_2) δ 53.26 (minor, 0.44 P), 45.40 (major, 0.56 P).

^{1}H NMR (500 MHz, CD_2Cl_2) δ 9.15 (s, 0.55H), 9.04 (s, 0.45H), 8.09 (d, *J* = 8.1 Hz, 1H), 7.98 (s, 3.1H), 7.88 (d, *J* = 5.5 Hz, 1.6H), 7.64 (d, *J* = 4.2 Hz, 1.7H), 7.53 (d, *J* = 6.8 Hz, 2.25H), 7.43 (m, 4.9 H), 7.34 (t, *J* = 7.0 Hz, 1.71), 7.31 – 7.24 (m, 1.6H), 7.24 – 7.08 (m, 5.6H), 6.91 (m, 1.6H), 5.37 (d, *J* = 7.9 Hz, 1.53H), 4.57 (d, *J* = 11.4 Hz, 1.1H), 4.05 (dd, *J* = 11.0, 3.9 Hz, 6H), -13.97 (t, *J* = 26.6 Hz, 1H), -16.71 (t, *J* = 19.9 Hz, 1H).

^{13}C NMR (101 MHz, CD_2Cl_2) δ 153.37 151.93, 144.21, 142.83, 136.58, 135.51, 134.46, 133.71, 133.65, 133.59, 133.35, 132.89, 130.76, 130.32, 130.09, 129.71, 129.45, 129.07, 128.87, 128.46, 128.08, 127.84, 127.50, 127.45, 127.11, 124.97, 124.45, 35.53 (t, J = 10.4 Hz), 34.91 (J = 8.0 Hz).

IR (thin film, KBr) = 1953 cm^{-1} (*minor*), 1932 cm^{-1} (*major*) (CO).

HR-ESI-MS m/z calcd. for $C_{40}H_{32}NONaP_2ClRu$ [8-Na]$^+$: 764.0589, found: 764.1075.

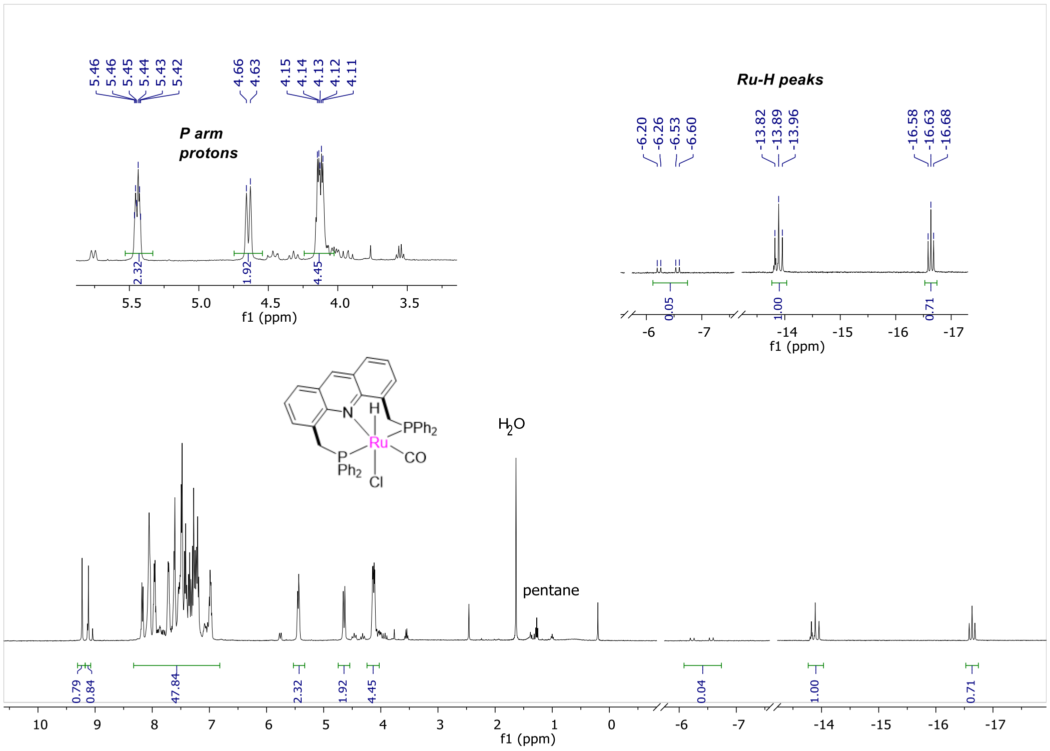

Figure S4. ^1H NMR spectrum (400 MHz) of Ru-Acr(Ph)HCl(CO) complex (**8**) in CD_2Cl_2.Two distinct hydride peaks of two different isomers are shown in inset.

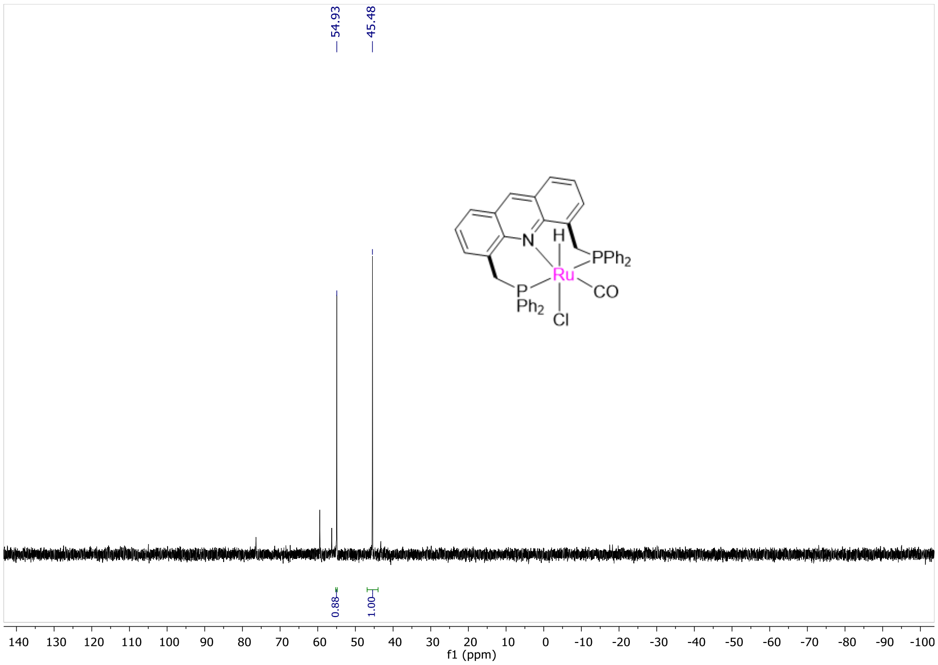

Figure S5. $^{31}P\{^{1}H\}$ NMR spectrum (162 MHz) of Ru-Acr(Ph)HCl(CO) complex (**8**) in CD_2Cl_2.Two distinct major isomers are seen, along with some minor complexes.

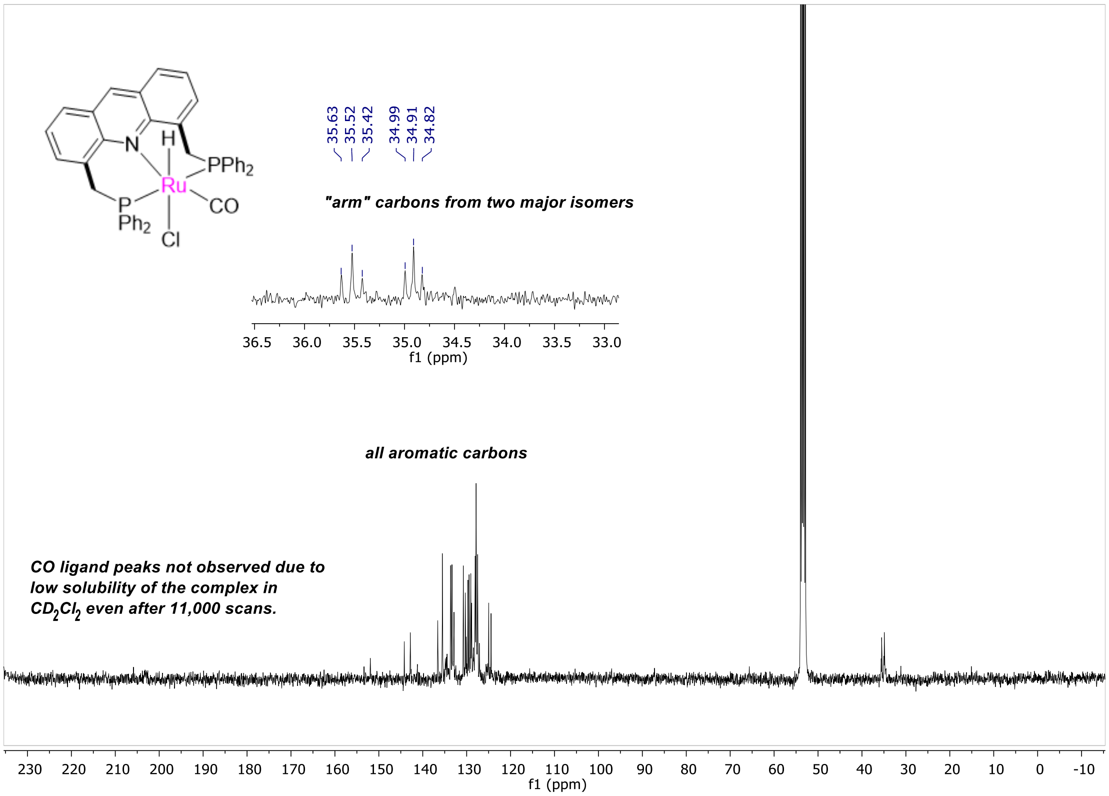

Figure S6. $^{13}C\{^{1}H\}$ NMR spectrum (101 MHz) of Ru-Acr(Ph)HCl(CO) complex (**8**) in CD_2Cl_2.

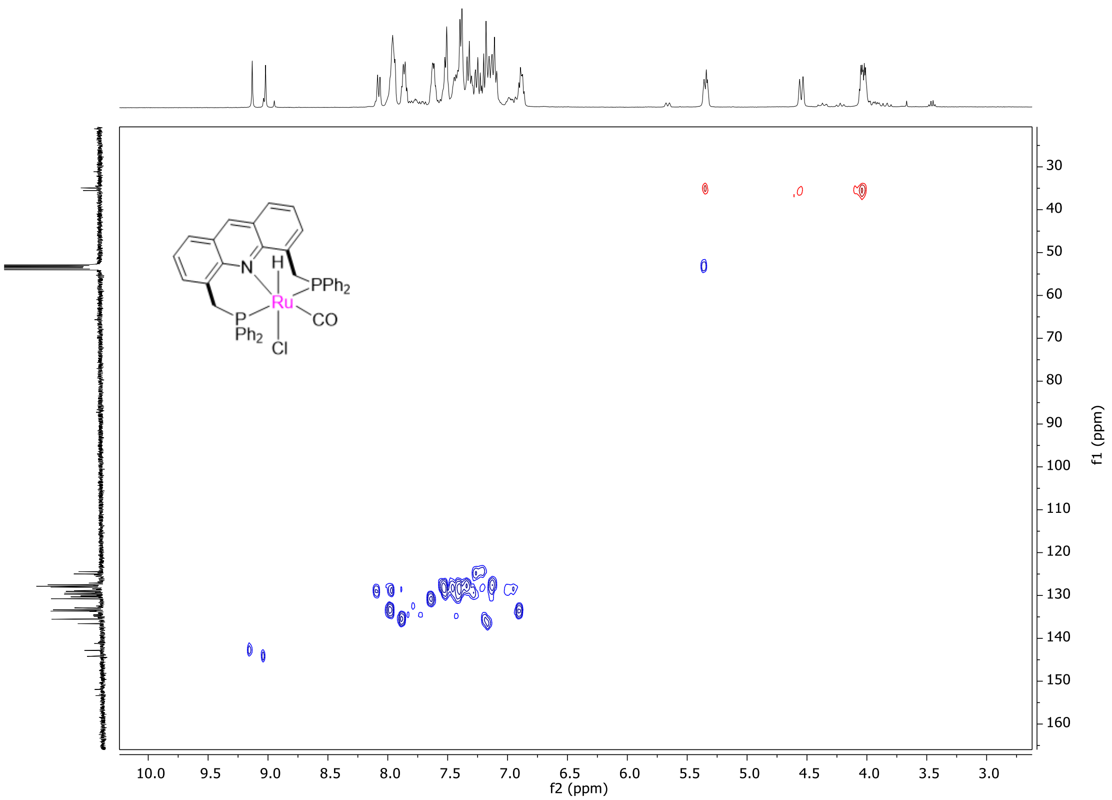

Figure S7. ^{1}H-^{13}C HSQC NMR spectrum of Ru-Acr(Ph)HCl(CO) complex (**8**) in CD_2Cl_2.

3. Standard procedure for furfural oxidation to furoic acid using water

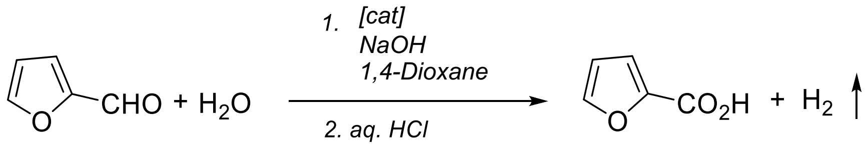

In a N_2 glove box, 10 µmol of ruthenium catalyst **6** (6 mg) was suspended in 2 mL of dry 1,4-dioxane in a 5 mL vial. To the solution, 1 mmol (96 mg) of furfural was added. The resulting mixture was transferred in a Schlenk flask. In another vial, 1.2 mmol (48 mg) of NaOH was weighed and added to the Schlenk flask. The flask was subsequently charged with 1 mL degassed water, taken out of the glove box, and dipped into a preheated oil bath (135 ^{o}C) with stirring. After given duration (48 h), the reaction solution was cooled to room temperature. The generated gas amount was measured in a gas burette which was further analyzed by GC. A known amount of mesitylene was added to the solution as an internal standard. A sample of the resulting solution was then analyzed by ^{1}H NMR (unlocked, relaxation delay 10 s). Yields of furoic acid salt and furfuryl alcohol were calculated from integration ratios between product aromatic proton peaks and mesitylene aromatic protons peak (δ ~ 6.4 ppm). The NMR solution was subsequently added to the parent solution and 1.5 mL of 4 M HCl was added. The organics were then extracted with ethyl acetate from the solution (2*3 mL), dried over $MgSO_4$, and the volatiles were removed to obtain furoic acid in 92% yield as white powder.

^{1}H NMR (300 MHz, DMSO-d_6) δ 7.90 (d, J = 1.8 Hz, 1H), 7.21 (d, J = 3.5 Hz, 1H), 6.64 (dd, J = 3.5, 1.8 Hz, 1H).

^{13}C NMR (75 MHz, DMSO-d_6) δ 159.80, 147.51, 145.31, 118.22, 112.58.

IR (thin film, KBr): 1692 cm^{-1}.

ESI-MS: 112.0

Reported data are in accordance with the previously reported values.[10]

3.1. ¹H NMR of selected crude reaction mixtures

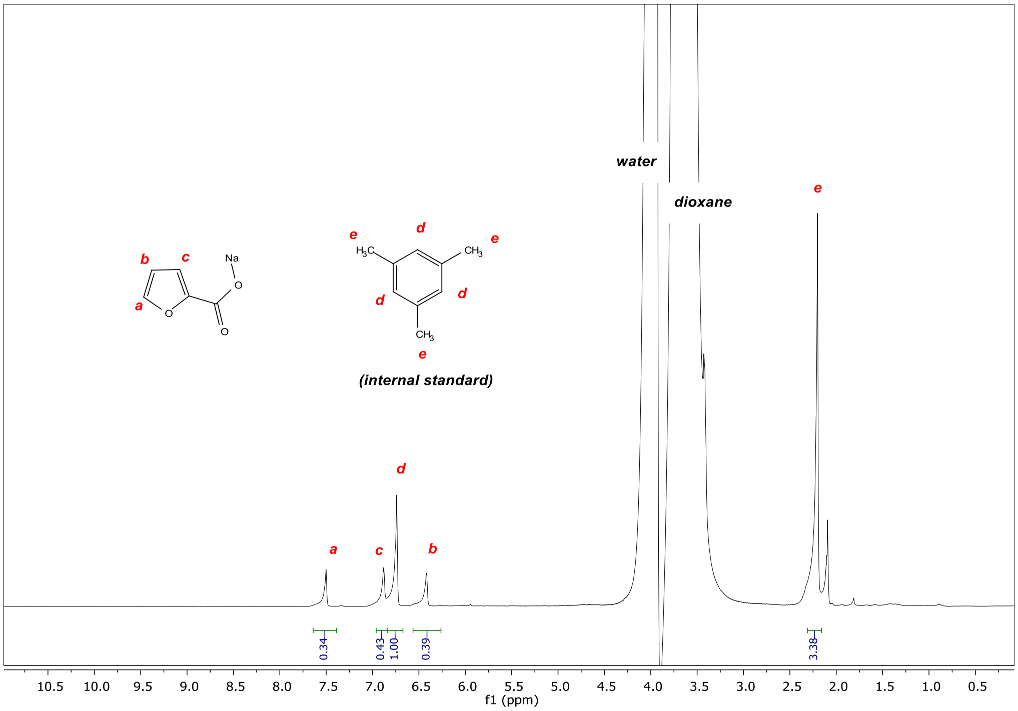

Figure S8. ¹H NMR spectrum of crude reaction mixture after complete oxidation of furfural to furoic acid salt.

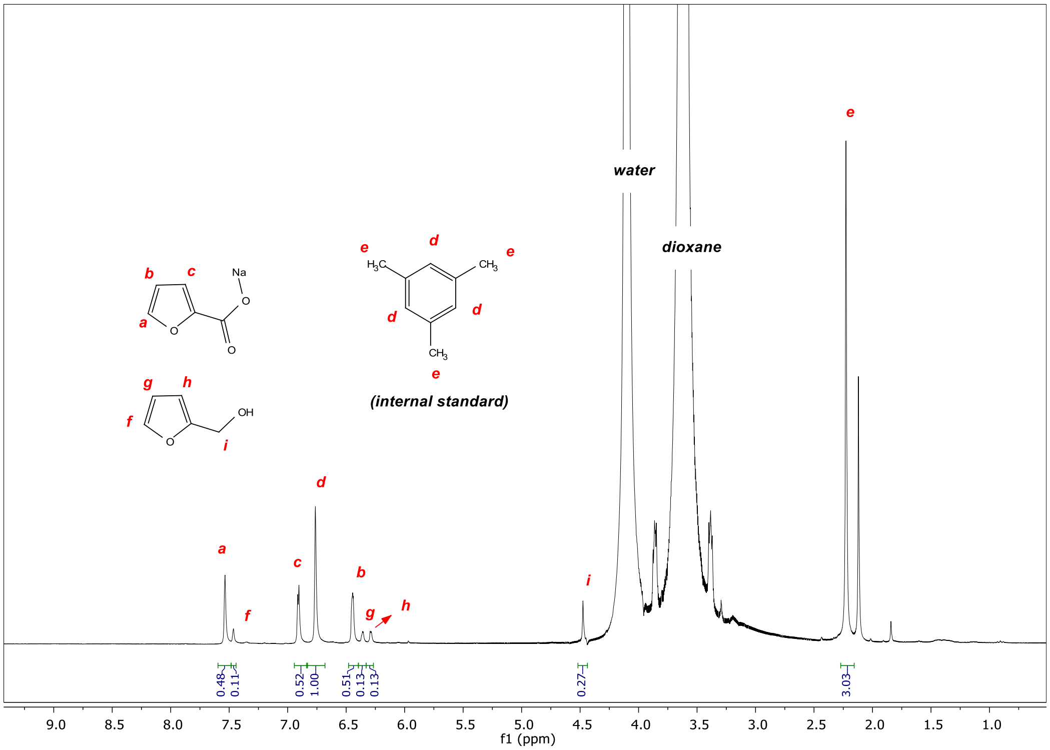

Figure S9. ¹H NMR spectrum of the crude reaction mixture after partial oxidation of furfural. Presence of furoic acid salt and furfuryl alcohol is seen in the solution.

3.2. ^{1}H and ^{13}C NMR of isolated furoic acid

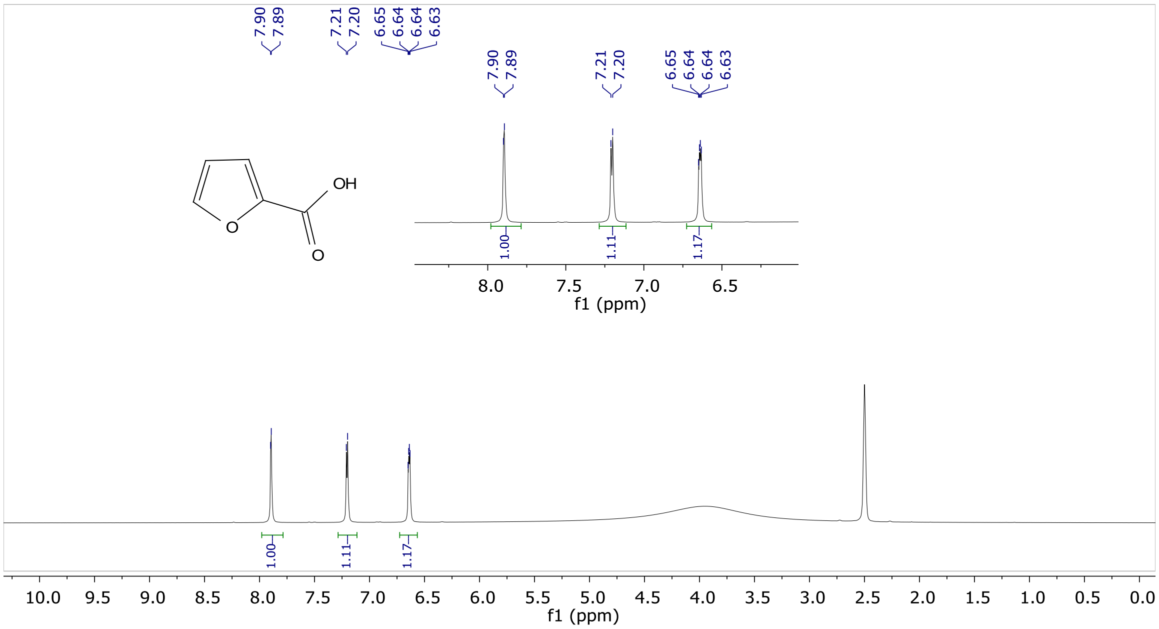

Figure S10. ^{1}H NMR spectrum (300 MHz) of isolated furoic acid in DMSO-d_6 after acid workup.

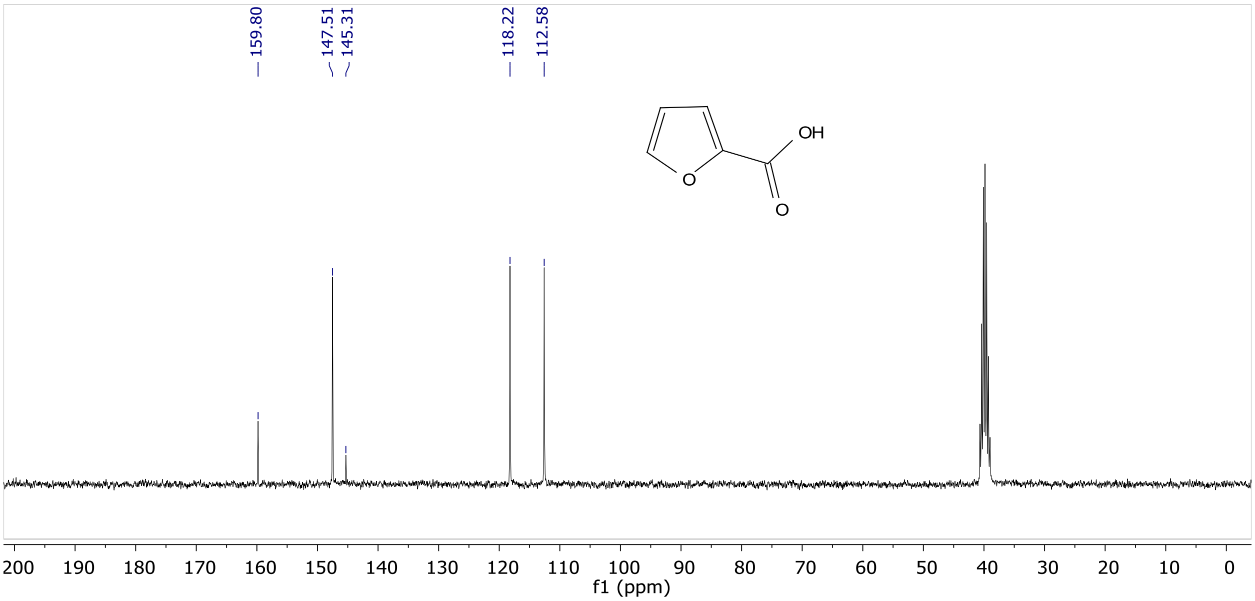

Figure S11. ^{13}C NMR spectrum (75 MHz) of isolated furoic acid in DMSO-d_6 after acid workup.

3.3. Extended data

Table S1. Furfural to furoic acid: Catalyst and condition screeing

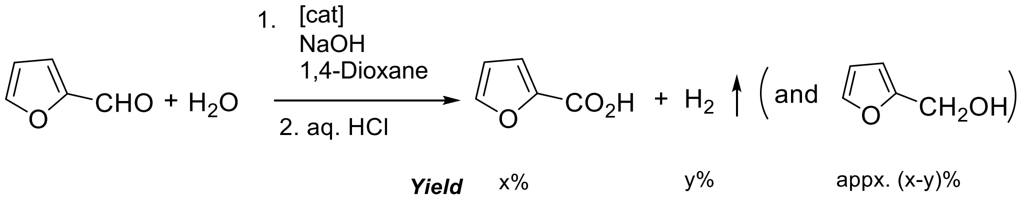

Screened Complexes as Pre-catalysts

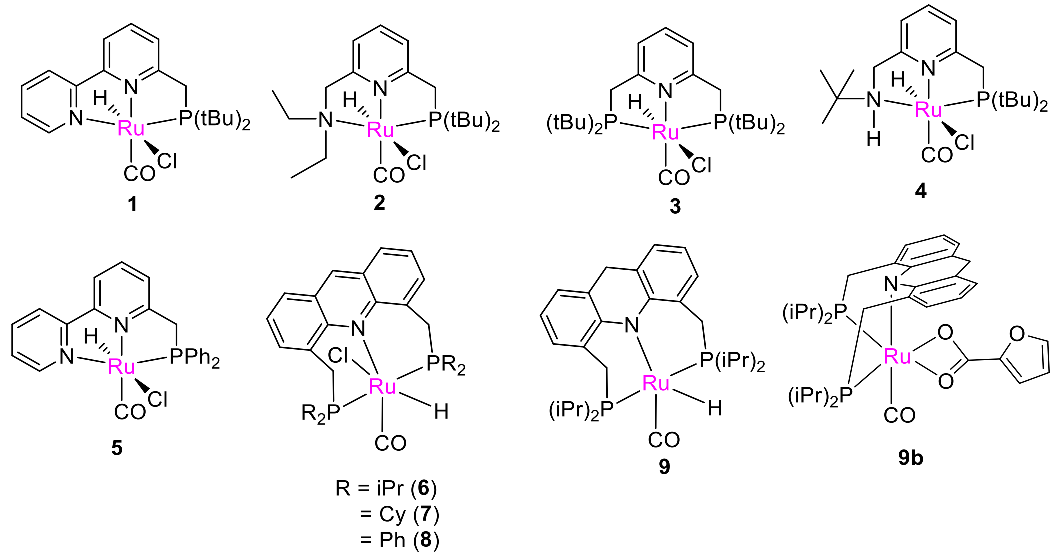

Entry	Cat (mol%)	NaOH (equiv.)	Conv. (%)[a]	H_2 (%)[b]	Acid (%)[a]	Alcohol (%)[a]
**1**	**1** (1)	1.2	>99	0	0	0
**2**	-	1.2	>99	0	0	0
**3**[c]	-	1.2	90	0	34	35
**4**	**2** (1)	1.2	99	0	0	0
**5**	**3** (1)	1.2	99	0	0	0
**6**	**4** (1)	1.2	99	0	0	0
**7**	**5** (1)	1.2	99	8	29	21
**8**	**6** (1)	1.2	99	80	87(80)	7
**9**	**6** (0.5)	1.2	99	42	72	20
**10**	**6** (0.25)	1.2	99	18	53	30
**11**	**6** (2)	1.2	99	85	92	5
**12**	**6** (1)	0.15	91	4	20	15

**13**	**6** (1)	0	65	0	0	0
**14**[d]	**6**(1)	1.2	99	95	97(92)	*
**15**[d]	**7**(1)	1.2	99	90	95	4
**16**[d]	**8**(1)	1.2	99	70	85	14
**17**[d]	**9**(1)	1.2	99	98	98	*
**18**[d]	**9b**(1)	1.2	99	96	97	2
**19**[d,e]	**6** (1)	1.2	99	61	83	13
**20**[d,f]	**6**(1)	1.2	99	20	61	37

Reaction conditions: furfural (1 mmol), NaOH (as specified), cat (as specified), 1,4-dioxane (2 mL), water (1 mL), 135 ^{o}C (bath temperature), 36 h. [a]Yields are based on ^{1}H NMR spectra with mesitylene as an internal standard [b]measured in a gas burette after reaction completion (error ±4%) [c]room temperature. [d]reaction time 48 h.[e]125 ^{o}C [f]115 ^{o}C *trace amount.

Table S2. Effect of base

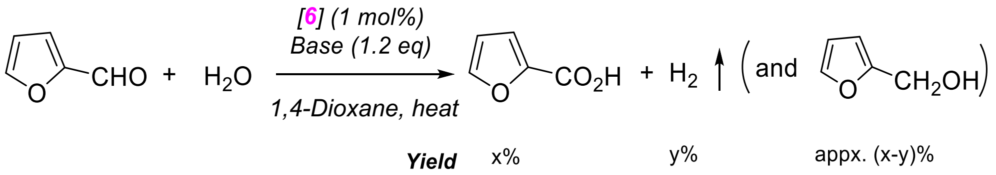

conjugate acid pKa in parenthesis

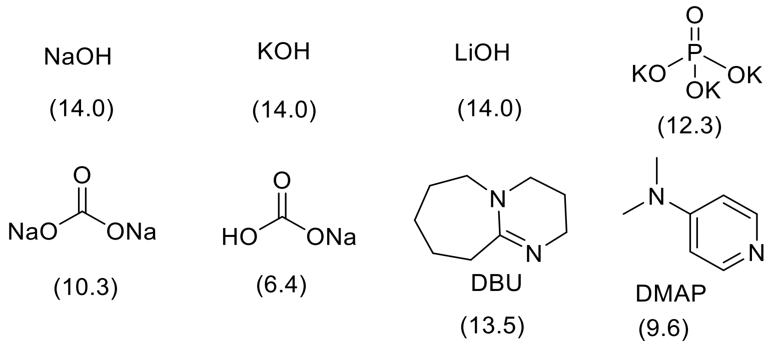

Entry	Cat (mol%)	Base (1.2 equiv.)	Conv. (%)[a]	H_2 (%)[a]	Acid (%)[a]	Alcohol (%)[a]
**1**	**6** (1)	NaOH	>99	95	97	0
**2**	**6** (1)	KOH	>99	90	94	3
**3**	**6** (1))	LiOH	>99	95	95	1
**4**	**6** (1)	K_3PO_4	>99	91	94	3
**5**	**6** (1)	Na_2CO_3	>99	82[b]	92	6
**6**	**6** (1)	$NaHCO_3$	99	-	52	40
**7**	**6** (1)	DBU	99	-	78	20
**8**	**6** (1)	DMAP	15	-	6	5

Reaction conditions. Furfural (1 mmol), base (1.2 mmol), **6** (1 mol%), water (1 mL), 1,4-dioxane (2 mL), 135 °C, 48 h. Reported yields are that of furoic acid salts prior to acidification. [a]Yields calculated as in Table S1 [b]generation of H_2/CO_2 gas mixture was observed.

4. Standard procedure for HMF oxidation to FDCA using water

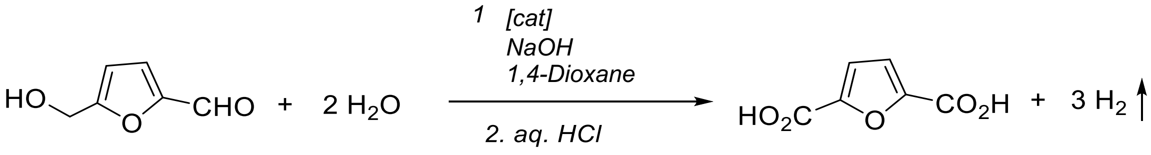

In a N_2 glove box, 10 μmol of ruthenium catalyst **6** (6 mg) was suspended in 2 mL of dry 1,4-dioxane in a 5 mL vial. To the solution, 0.5 mmol (63 mg) of HMF was added. The resulting mixture was transferred to a Schlenk flask. In another vial, 1.1 mmol (44 mg) of NaOH was added to the Schlenk flask. The flask was subsequently charged with 1 mL degassed water, taken out of the glove box, and subsequently dipped into a preheated oil bath (160 °C) with stirring. After the given duration (68 h), the reaction solution was cooled to room temperature. The generated gas amount was measured in a gas burette which was further analyzed by GC. A known amount of mesitylene was added to the solution as an internal standard. A sample of the resulting solution was then analyzed by ^{1}H NMR (unlocked, relaxation delay 10 s). Yields of FDCA salt and other products was calculated from integration ratios between product aromatic proton peaks and mesitylene aromatic protons peak (δ ~ 6.4 ppm). The NMR solution was subsequently added to the parent solution, 1,4-dioxane was removed *in vacuo*, and 1.5 mL of cold 4 M HCl was added to the solution. Formation of white precipitates were observed which were filtered off, washed with ether, cold water, hexane and dried to obtain FDCA in 89% yield as a white powder.

^{1}H NMR (300 MHz, DMSO-d_6) δ 7.28 (s, 1H).

^{13}C{^{1}H} NMR (75 MHz, DMSO-d_6) δ 159.36, 147.45, 118.90.

IR (thin film, KBr): 1700 cm^{-1}.

Reported data are in accordance with the previously reported values.[11]

4.1. ^{1}H NMR of selected crude reaction mixtures

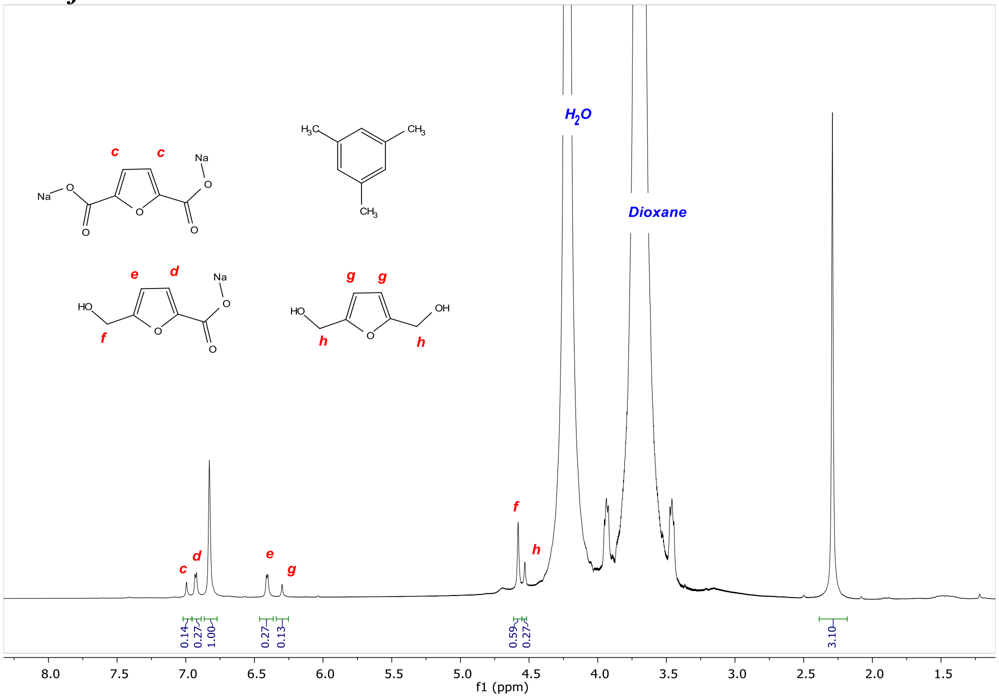

Figure S12. ^{1}H NMR of the crude reaction mixture after incomplete oxidation of HMF to FDCA (Figure 2G, entry 1) showing the formation of HMFA as an intermediate. Bis(hydroxymethyl)furan is also observed in the solution (peak g and h).

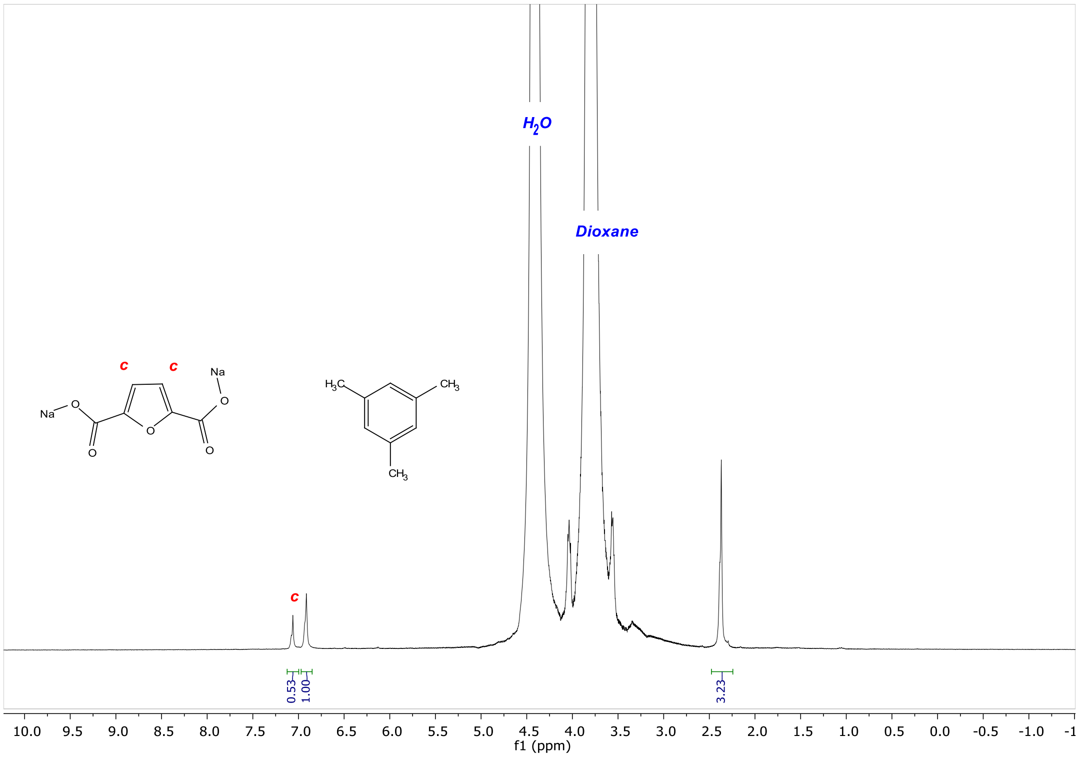

Figure S13. ^{1}H NMR of the crude reaction mixture after complete oxidation of HMF to FDCA disodium salt. (Figure 2G, entry 5).

4.2. ^{1}H NMR of isolated FDCA

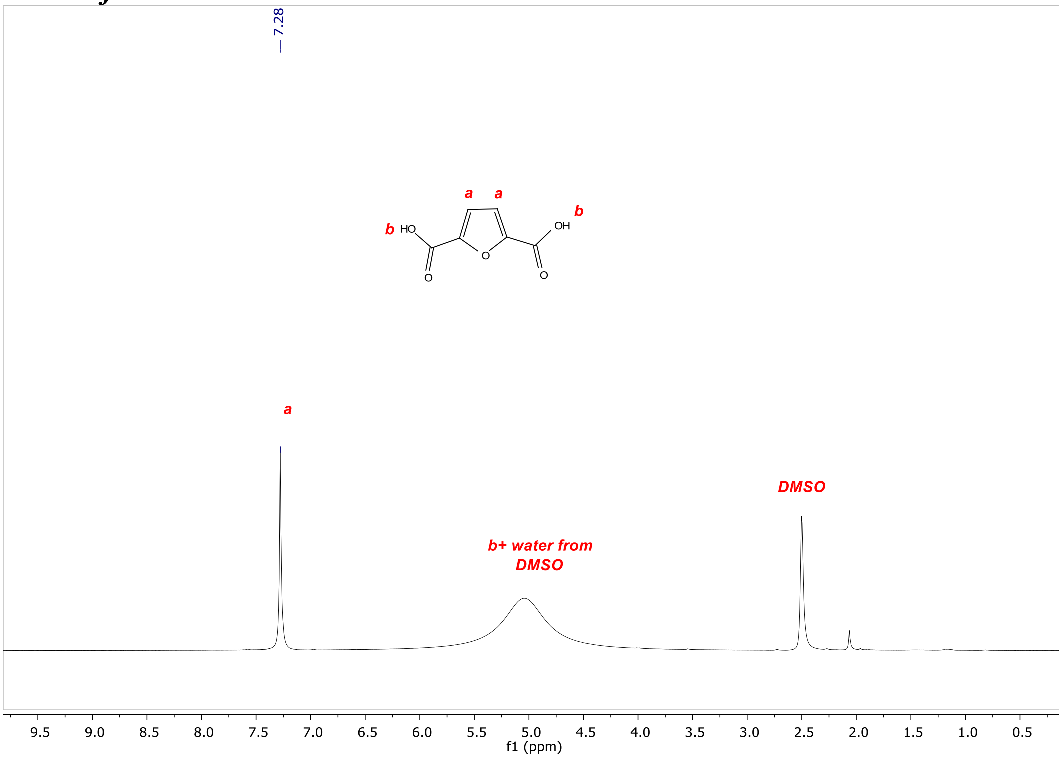

Figure S14. ^{1}H NMR spectrum of isolated FDCA after acid workup in DMSO-d_6.

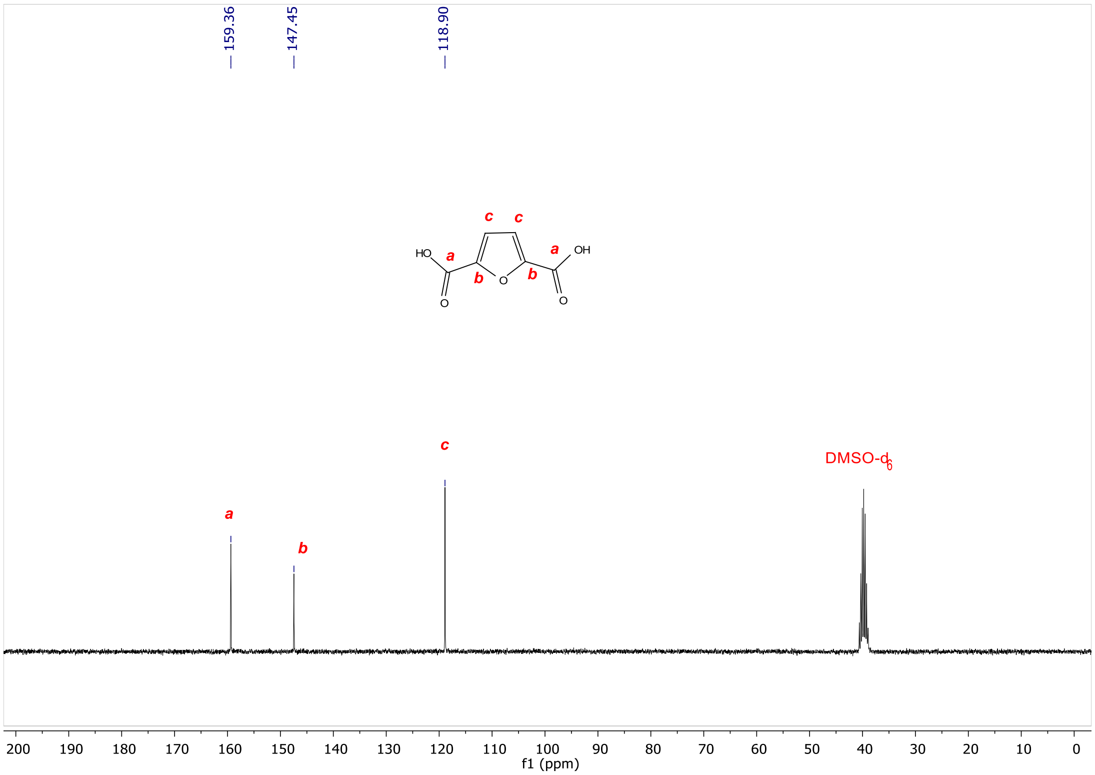

Figure S15. ^{13}C NMR spectrum of isolated FDCA after acid workup in DMSO-d_6.

5. Procedures for additional experiments

5.1. Procedure for H_2 evolution profile experiment

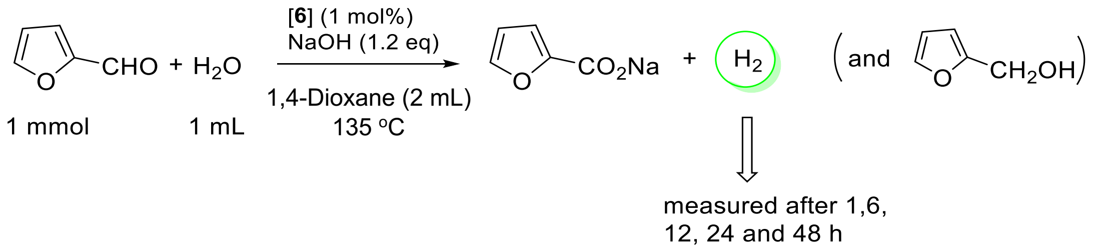

In a N_2 glove box, 10 µmol of ruthenium catalyst **6** (6 mg) was suspended in 2 mL of dry 1,4-dioxane in a 5 mL vial. To this, 1 mmol (96 mg) of furfural was added. The resulting mixture was transferred to a Schlenk flask. In another vial, 1.2 mmol (48 mg) of NaOH was weighed and added to the Schlenk flask. The flask was charged with 1 mL degassed water, taken out of the glove box, and dipped into a preheated oil bath (135 ºC) with stirring. After given intervals (15 min, 1 h, 6 h, 12 h, 24 h and 48 h), the reaction solution was cooled to room temperature. and generated gas amount was measured in a gas burette. The graph obtained between time and cumulative H_2 generation is shown below.

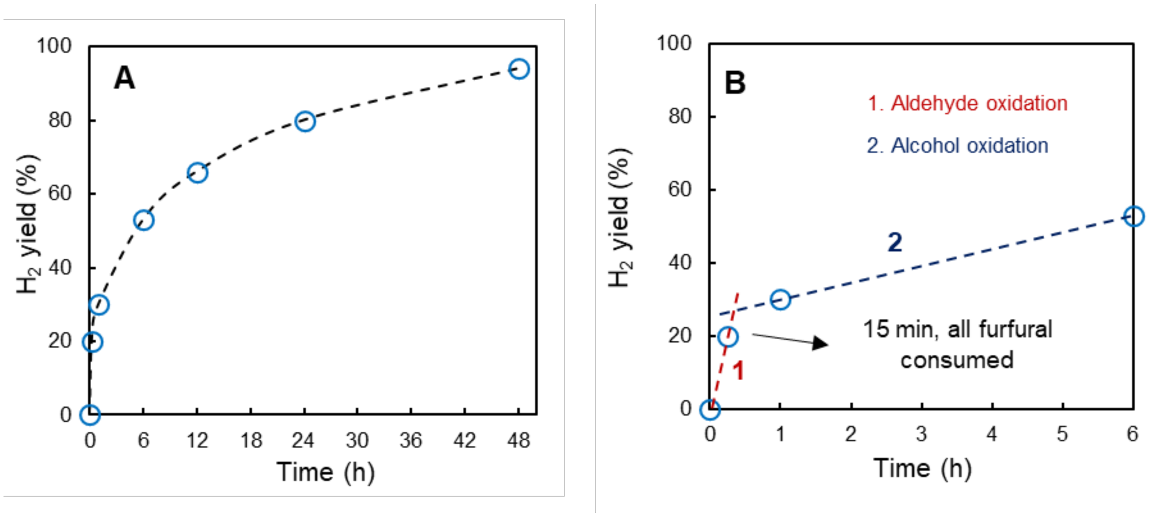

Figure S16. (A) H_2 evolution profile with time from the reaction mixture. (B) enlarged version of first 6 h gas evolution distinctly showing faster aldehyde dehydrogenation rate compared to the alcohol dehydrogenation.

5.2. Procedure for control experiment without catalyst

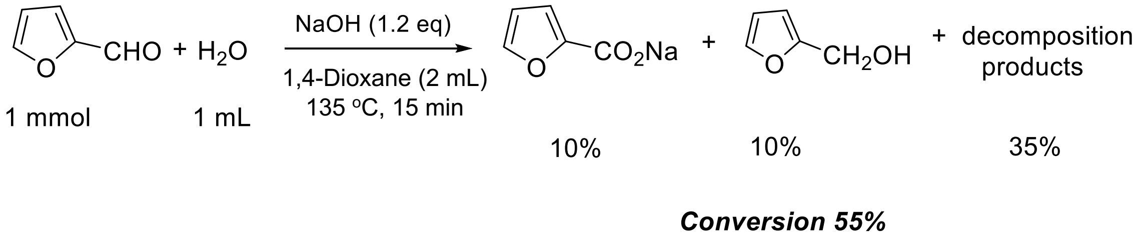

In a N_2 glove box, 1 mmol (96 mg) of furfural and 0.5 mmol of mesitylene (60 mg) were dissolved in 2 mL 1,4-dioxane which was added to a Schlenk flask. In another vial, 1.2 mmol (48 mg) of NaOH was weighed and subsequently added to the Schlenk flask. The flask was then charged with 1 mL degassed water, taken out of the glove box, and dipped into a preheated oil bath (135 ^{o}C) with stirring. After 15 min, the solution turned dark brown color. The flask was taken out of the oil bath and cooled to room temperature. A portion of the sample was subsequently analyzed by ^{1}H NMR (unlocked) and GC-MS.

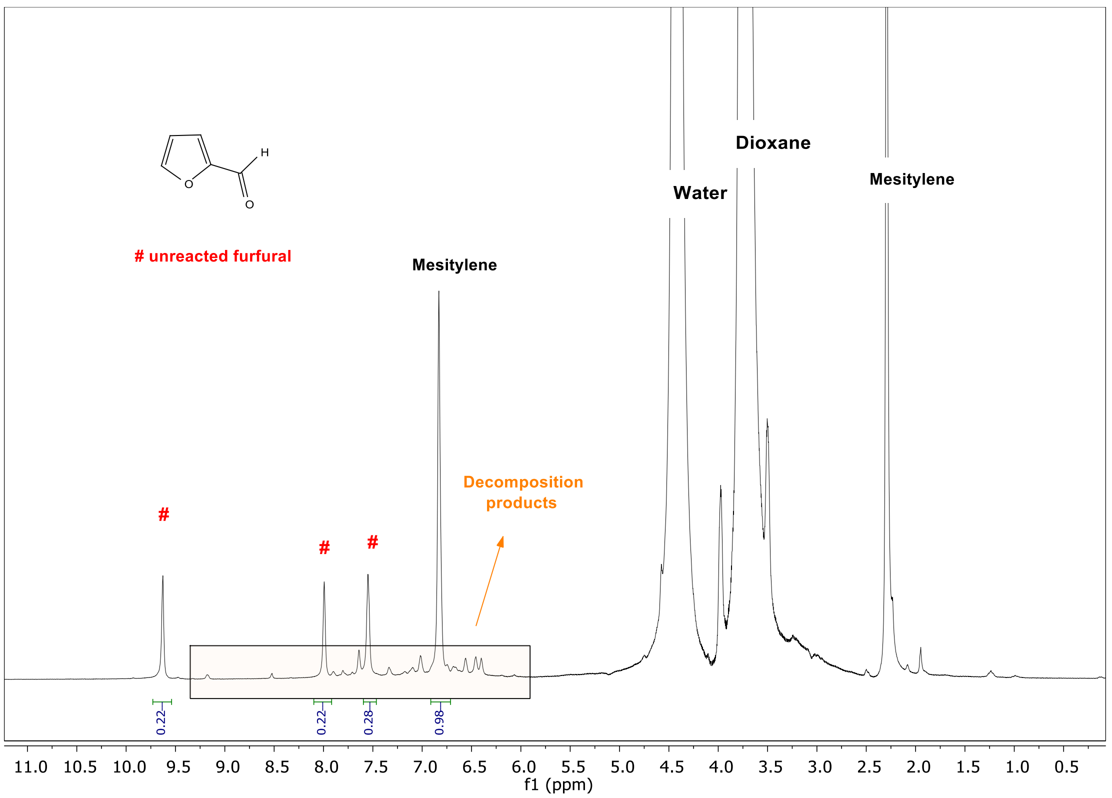

Figure S17. ^{1}H NMR spectrum after the reaction. Unreacted furfural is seen along with decomposition products including furoic acid salt (10%), furfuryl alcohol (10%), formate salt (2%) and other unknown products.

5.3. Procedure for control experiment with catalyst

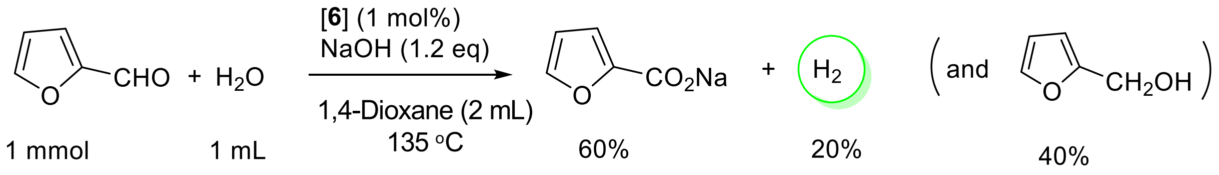

In a N_2 glove box, 0.01 mmol of complex **6**, 1 mmol (96 mg) of furfural and 0.5 mmol of mesitylene (60 mg) were mixed in 2 mL 1,4-dioxane which was added to a Schlenk flask. In another vial, 1.2 mmol (48 mg) of NaOH was weighed and subsequently added to the Schlenk flask. The flask was then charged with 1 mL degassed water, taken out of the glove box, and subsequently dipped into a preheated oil bath (135 °C) with stirring. After 15 min, the flask was taken out of the oil bath and cooled to room temperature. A portion of the sample was subsequently analyzed by ^{1}H NMR (unlocked) and GC-MS.

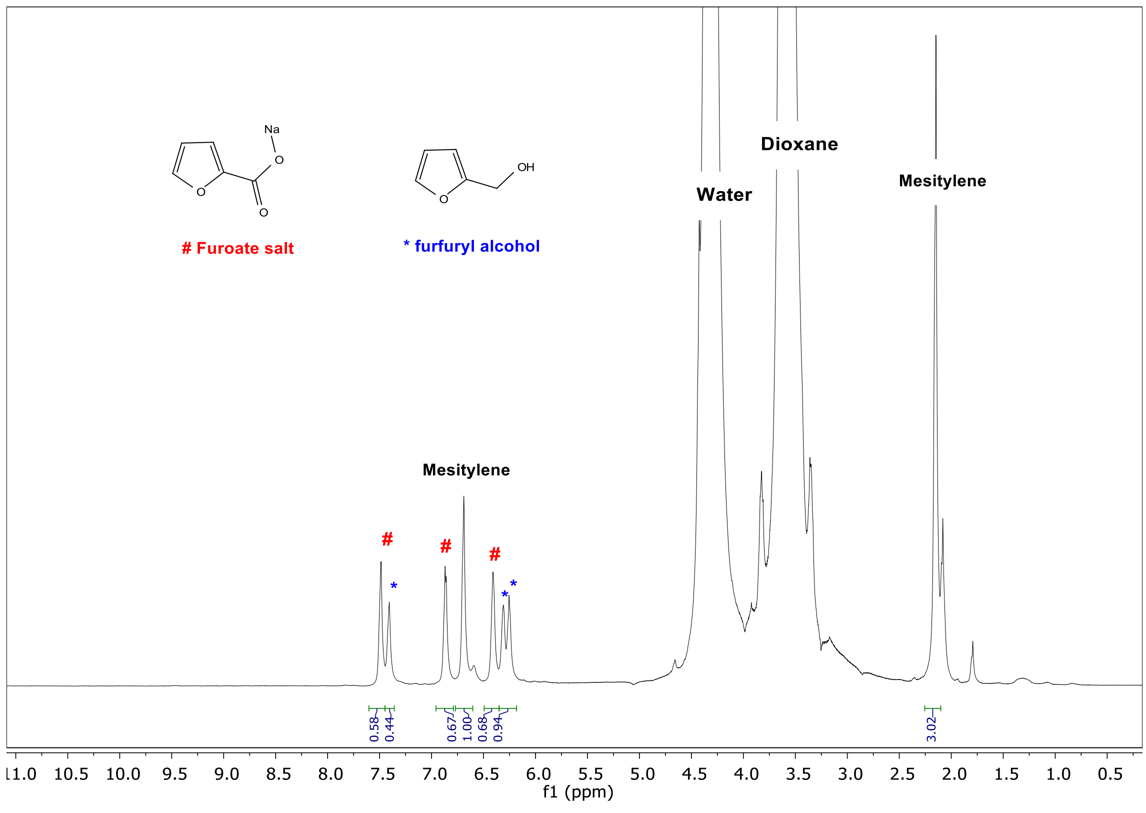

Figure S18. ^{1}H NMR spectrum after the reaction. Complete consumption of furfural is seen along with selective generation of furoic acid salt (60%) and furfuryl alcohol (40%). No decomposition was observed.

5.4. Procedure for Tishchenko coupling

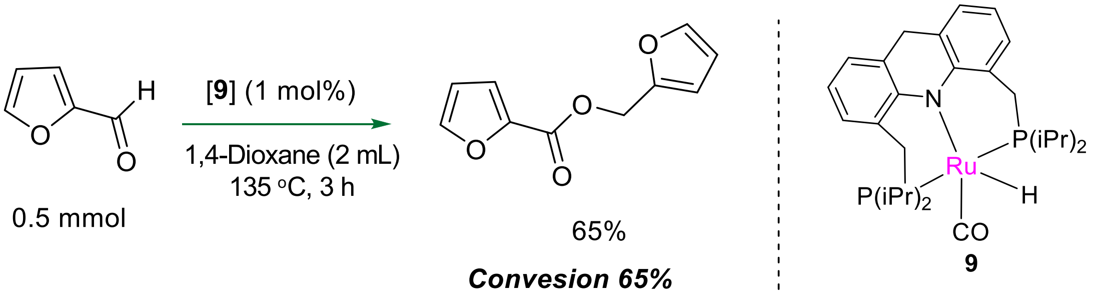

In a N_2 glove box, 5 µmol of complex **9** and 0.5 mmol (48 mg) of furfural were dissolved in 2 mL 1,4-dioxane which was added to a Schlenk flask. The flask taken out of the glove box and subsequently dipped into a preheated oil bath (135 °C) with stirring. After 3 h, the solution was cooled to room temperature. A know amount of mesitylene was added to the solution and it was analyzed by ^{1}H NMR and GC-MS.

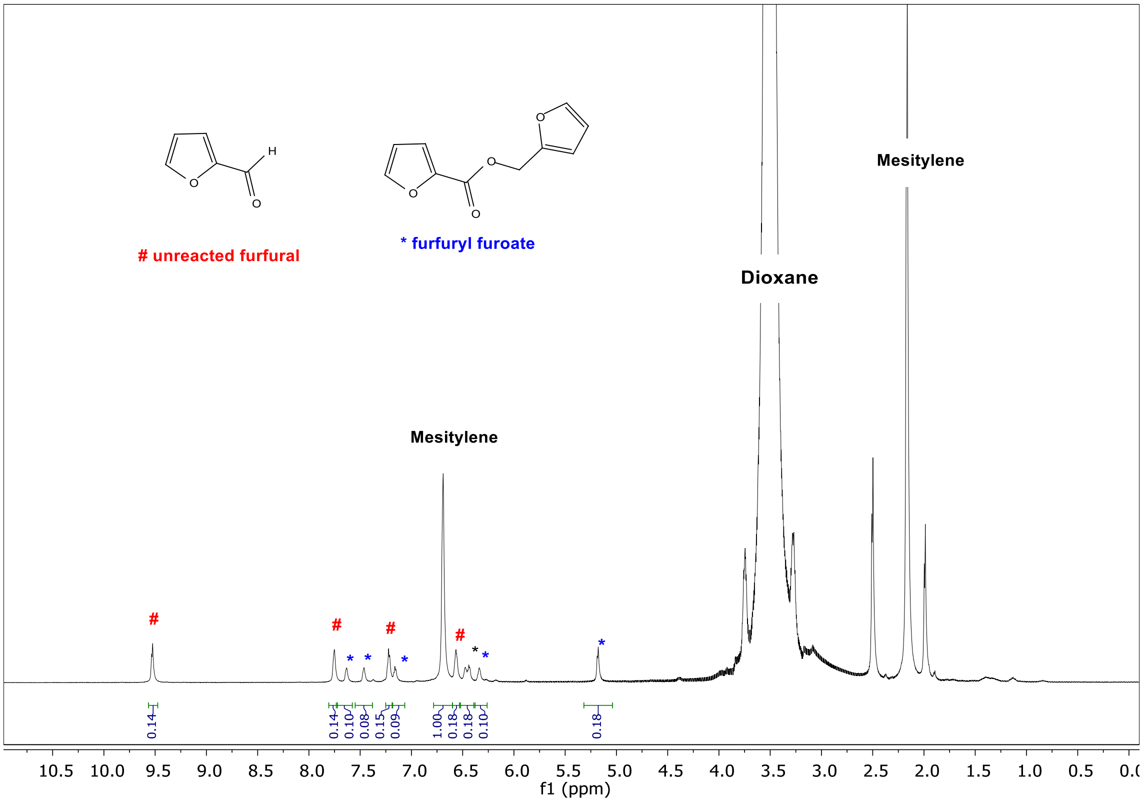

Figure S19. ^{1}H NMR spectrum after the reaction. Selective generation of furfuryl furoate was observed in 65% yield, the rest being unreacted furfural.

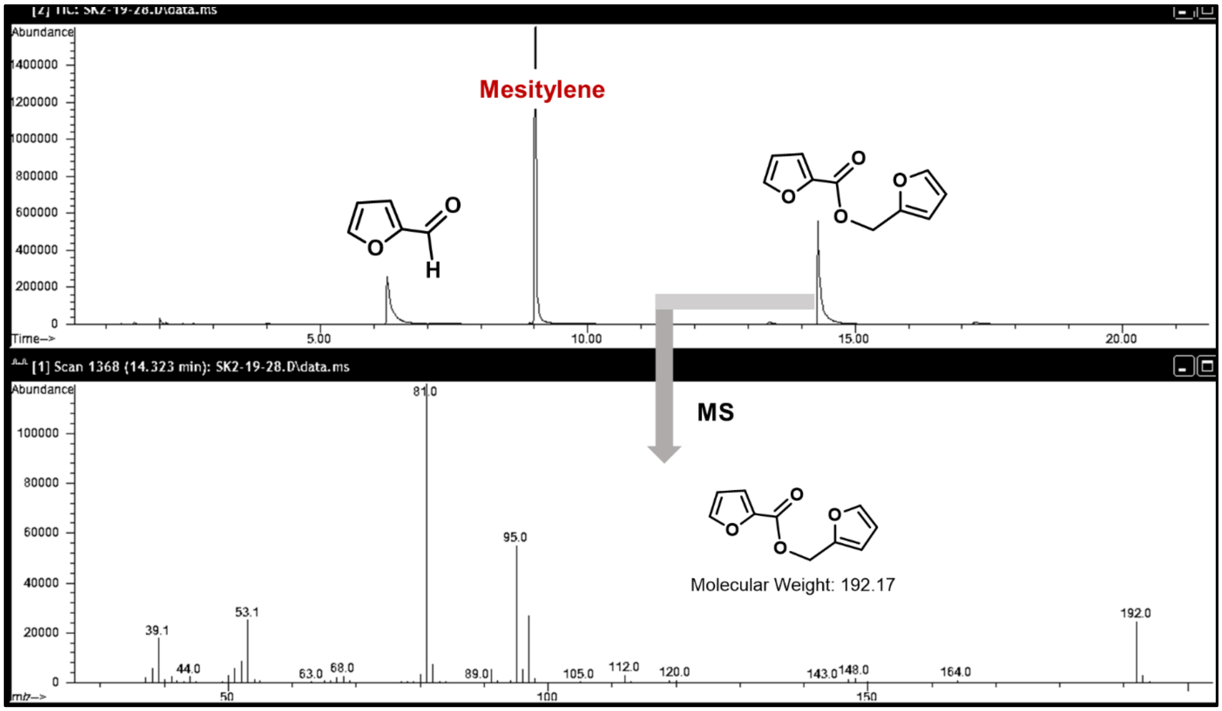

Figure S20. GC-MS chromatogram after the reaction. Top panel shows the selective generation of product from furfural. Bottom panel shows the mass fragmentation pattern of the product which is identified as furfuryl furoate.

5.5. Procedure of the catalyst recycling study

In a N_2 glove box, 0.02 mmol of complex **6**, 1 mmol (96 mg) of furfural and 0.5 mmol of mesitylene (60 mg) were mixed in 2 mL 1,4-dioxane which was added to a Schlenk flask. In another vial, 1.2 mmol (48 mg) of NaOH was weighed and subsequently added to the Schlenk flask. The flask was then charged with 1 mL degassed water, taken out of the glove box, and subsequently dipped into a preheated oil bath (135 ^{o}C) with stirring. After 36 h, the flask was taken out of the oil bath and cooled to room temperature. The flask was then taken inside a glove box and a portion of the sample (0.5 mL) was subsequently analyzed by ^{1}H NMR (unlocked) to determine the furoate yield. The NMR solution was added back to the flask and all solvents were removed subsequently *in vacuo* to obtain a solid containing furoate salt and the catalyst. The catalyst was then extracted with benzene (3 x 1 mL). The organic solutions were combined and removal of benzene from the solution recovered the active catalyst, which was used for the next cycle with a fresh batch of the other reagents (furfural, NaOH, water and 1,4-dioxane).

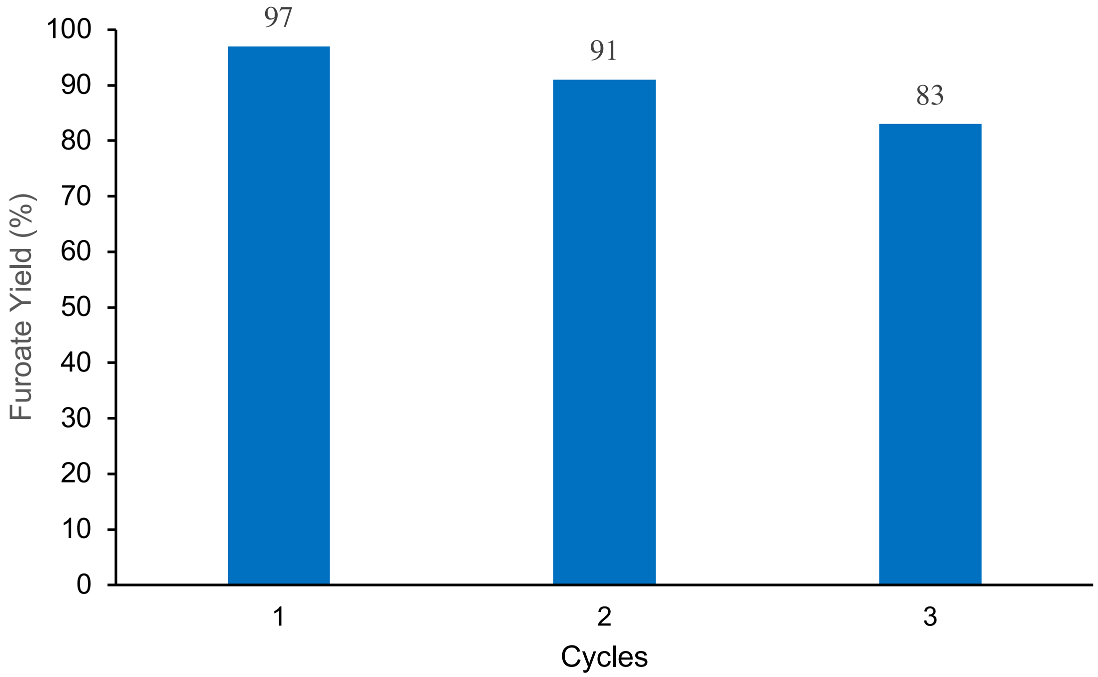

Figure S21. Yield of sodium furoate in subsequent cycles.

5.6. Procedure for gram scale reaction

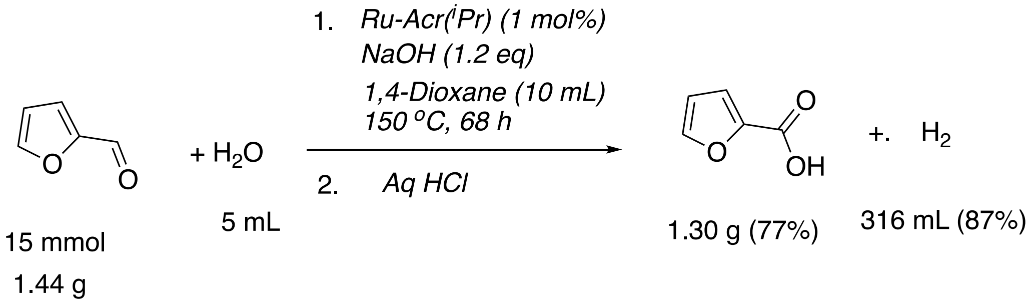

In a N_2 glove box, 0.15 mmol of ruthenium catalyst **6** (90 mg) was suspended in 10 mL of dry 1,4-dioxane in a 20 mL vial. To this, 15 mmol (1.44 g) of furfural was added. The resulting mixture was transferred to a 100 mL Schlenk flask. In another vial, 18 mmol (720 mg) of NaOH was weighed and added to the Schlenk flask. The flask was charged with 5 mL degassed water, taken out of the glove box, and dipped into a preheated oil bath (150 °C) with stirring. At given intervals (1 h, 5 h, 24 h and 68 h), the reaction solution was cooled to room temperature and the generated gas amount was measured in a gas burette. H_2 yield was calculated based on the overall gas collected. After 68 h, the flask was opened and 10 mL of 4 M HCl was added to the solution. The organics were then extracted with ethyl acetate from the solution (3*20 mL), dried over $MgSO_4$, and the volatiles were removed to obtain furoic acid in 77% yield (1.30 g) as white powder.

5.7. Procedure for BHMF oxidation using water

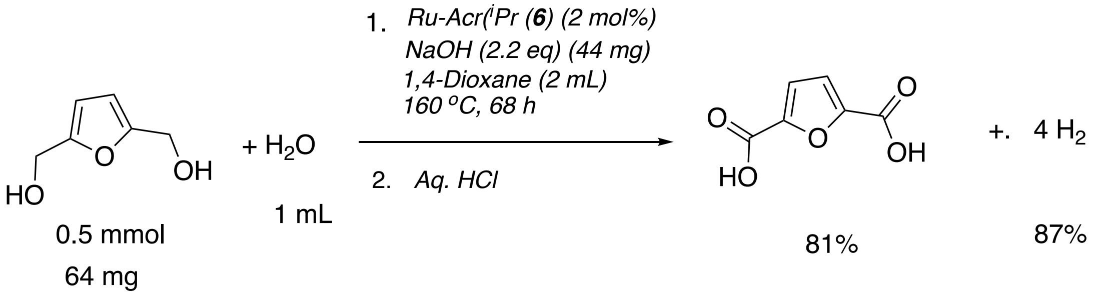

In a N_2 glove box, 10 μmol of ruthenium catalyst **6** (6 mg) was suspended in 2 mL of dry 1,4-dioxane in a 5 mL vial. To the solution, 0.5 mmol (64 mg) of BHMF was added. The resulting mixture was transferred to a Schlenk flask. In another vial, 1.1 mmol (44 mg) of NaOH was added to the Schlenk flask. The flask was subsequently charged with 1 mL degassed water, taken out of the glove box, and subsequently dipped into a preheated oil bath (160 °C) with stirring. After the given duration (68 h), the reaction solution was cooled to room temperature. The generated gas amount was measured in a gas burette which was further analyzed by GC. Subsequently, 1.5 mL of cold 4 M HCl was added to the solution. Formation of white precipitates were observed which were filtered off, washed with ether, cold water, hexane and dried to obtain FDCA in 81% yield as a white powder.

6. Mechanistic studies

6.1. Synthesis and characterization of the furoate complex 9b

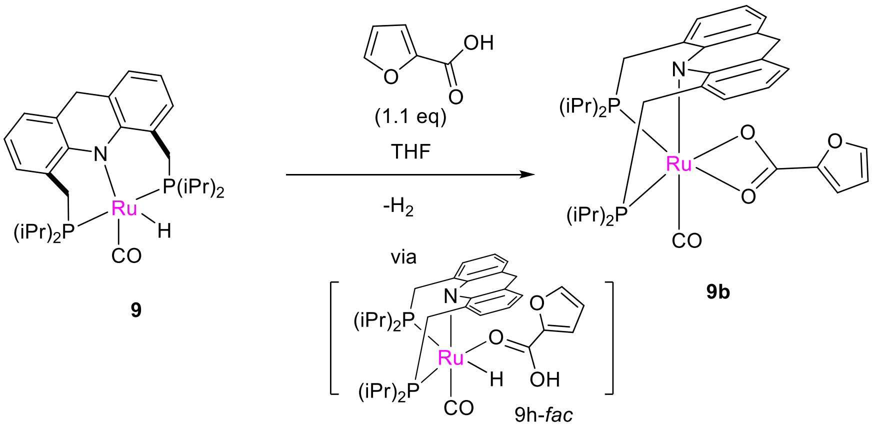

Procedure: Complex **9** (12 mg) was dissolved in 3 mL of THF in a 20 mL vial. To this solution, 1.1 equiv. of furoic acid, pre-dissolved in 2 mL THF, was added dropwise. The resulting solution was stirred for 1 hour. THF was subsequently removed in vacuo, and the remaining solid was dissolved 5 mL benzene. Afterwards, the benzene solution was filtered through a short Celite pad, and benzene was removed *in vacuo* to afford complex **9b** as a yellow orange solid (10 mg; 76%).

$^{31}P\{^{1}H\}$ NMR (162 MHz, Benzene-d_6) δ 88.40.

^{1}H NMR (400 MHz, Benzene-d_6) δ 7.09 (t, J = 4.5 Hz, 1H), 6.99 (s, 2H), 6.98 (s, 2H), 6.74 (d, J = 3.4 Hz, 1H), 6.72 (d, J = 1.6 Hz, 1H), 5.69 (m, 1H), 3.84 (d, J = 16.7 Hz, 1H), 3.70 (d, J = 16.7 Hz, 1H), 2.80 (dd, J = 14.3, 12.3 Hz, 1H), 2.55 (dd, J = 12.7, 6.5 Hz, 2H), 2.03 (h, J = 7.5 Hz, 3H), 1.44 – 1.37 (m, 4H), 1.17 – 1.08 (m, 4H), 1.07 – 0.94 (m, 8H).

^{13}C NMR (101 MHz, Benzene-d_6) δ 202.41 (t, J = 15.4 Hz), 175.50, 152.98, 147.47, 144.37, 129.67, 127.17, 126.15, 121.94, 118.43, 115.17, 110.78, 33.99, 29.83 – 28.97 (m), 28.30 – 27.75 (m), 26.28 – 25.41 (m), 20.25, 19.38, 18.75, 18.53 (t, J = 3.3 Hz).

IR (thin film, KBr) = 1935 cm^{-1} (ν_{CO}), 1595 cm^{-1} ($\nu_{as}COO$), 1568 cm^{-1} ($\nu_{s}COO$).

HR-ESI-MS m/z calcd. for $C_{33}H_{43}NO_4NaP_2Ru$ [**9b**-Na]$^{+}$: 704.1609, found: 704.0860.

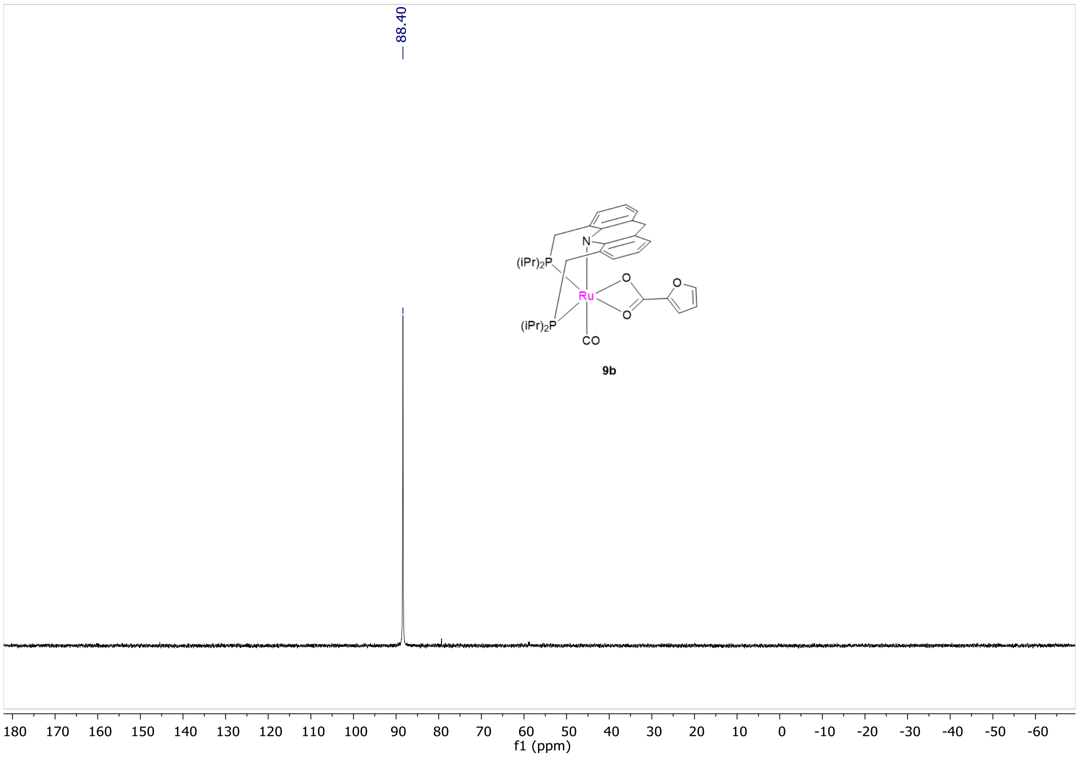

Figure S22. $^{31}P\{^{1}H\}$ (162 MHz) NMR spectrum of complex **9b** in C_6D_6.

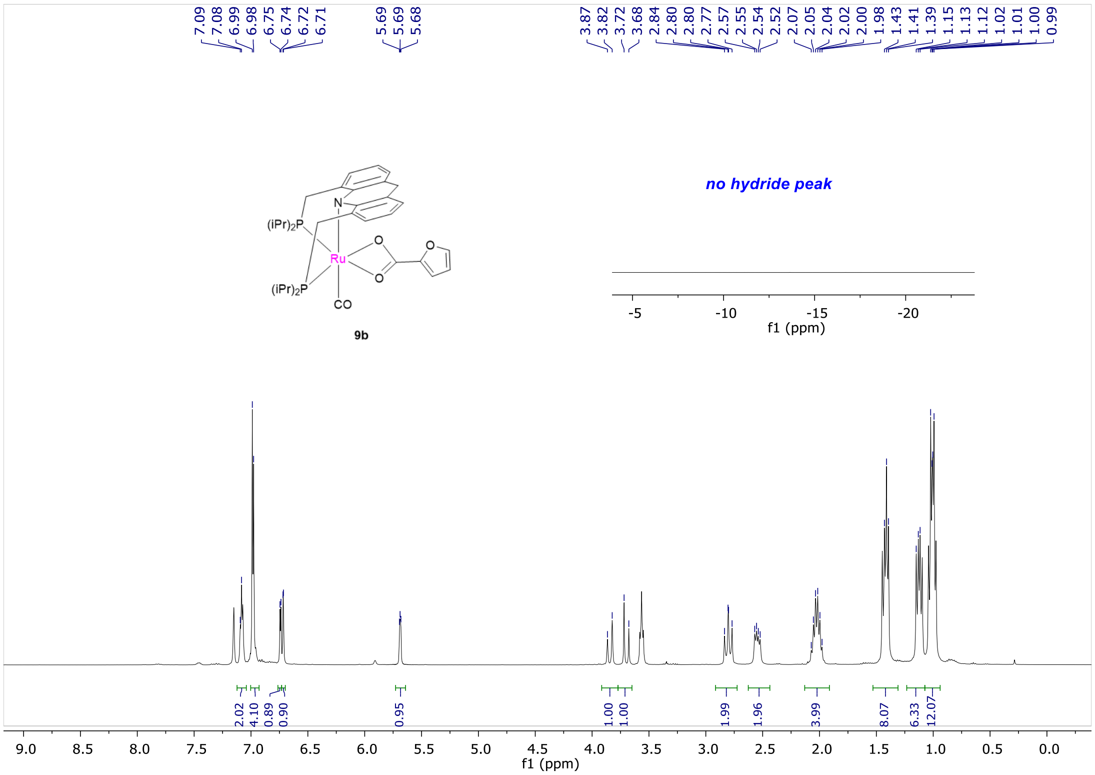

Figure S23. ^{1}H NMR spectrum (400 MHz) of complex **9b** in C_6D_6.

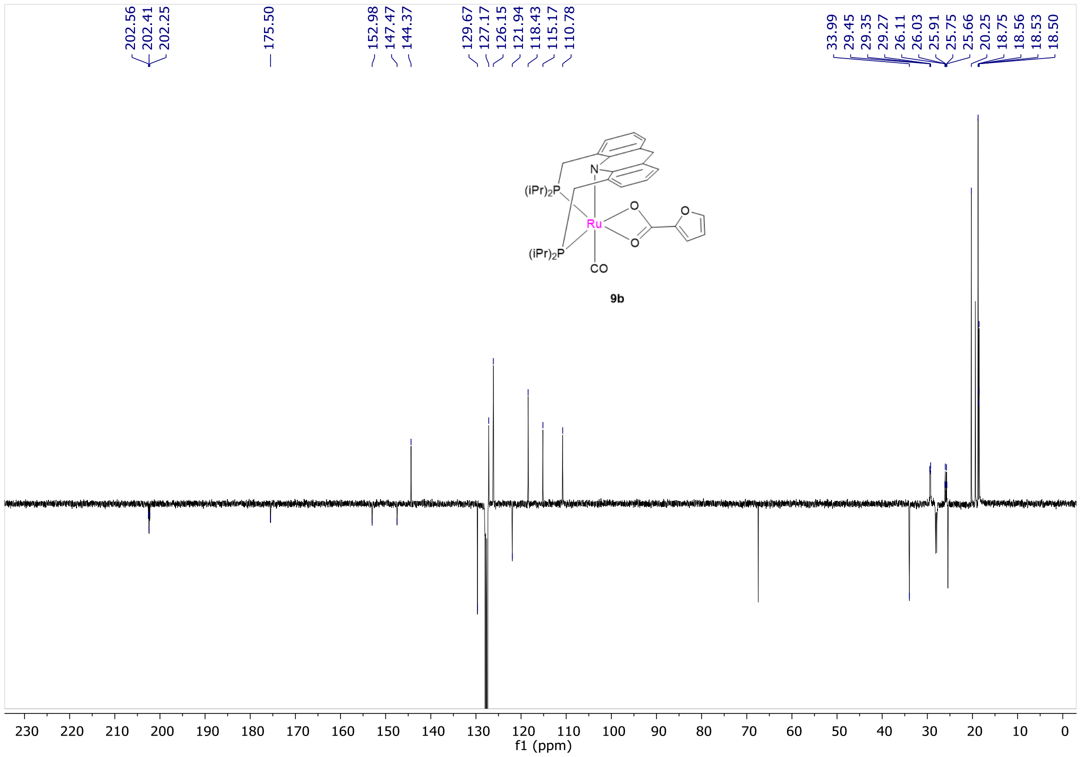

Figure S24. DEPT-135 NMR spectrum of complex **9b** in C_6D_6.

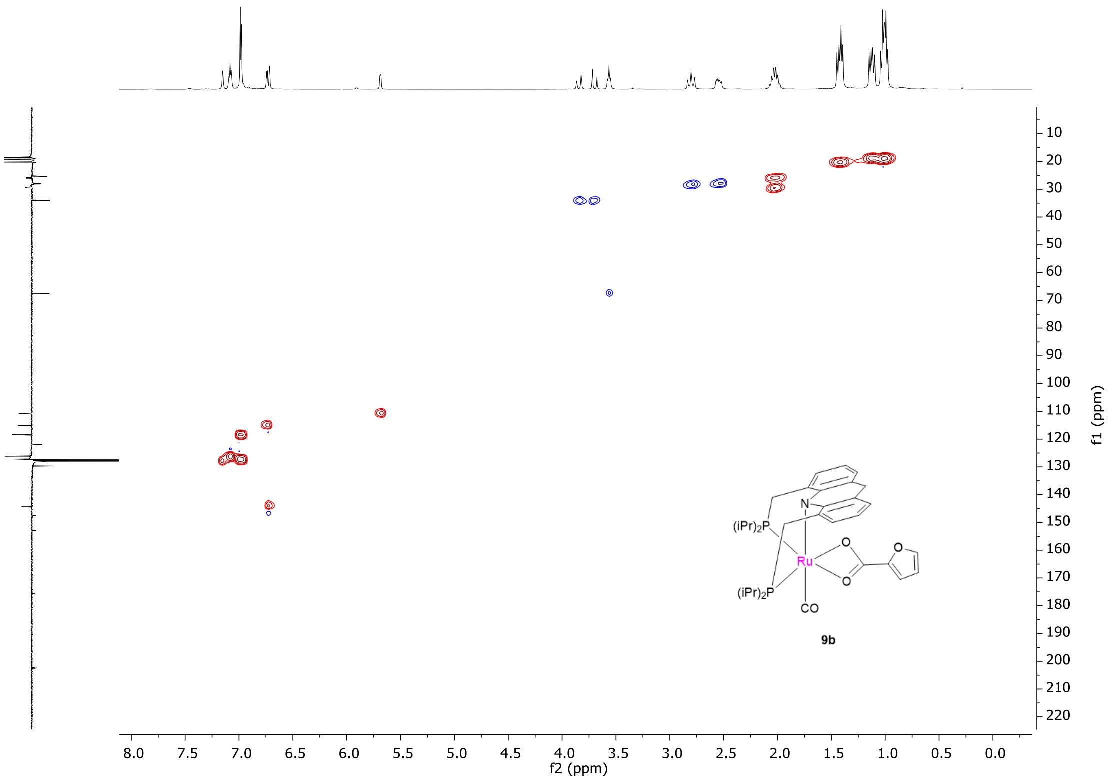

Figure S25. ^{1}H-^{13}C HSQC NMR spectrum of complex **9b** in C_6D_6.

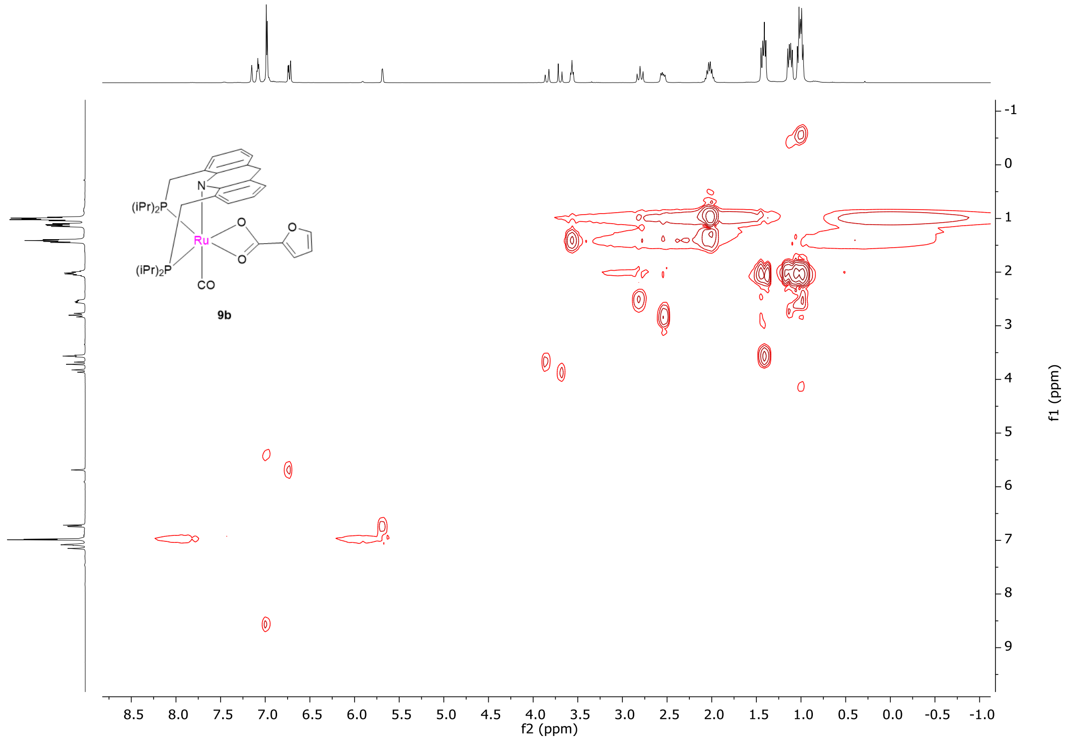

Figure S26. ^{1}H-^{1}H COSY NMR spectrum of complex **9b** in C_6D_6.

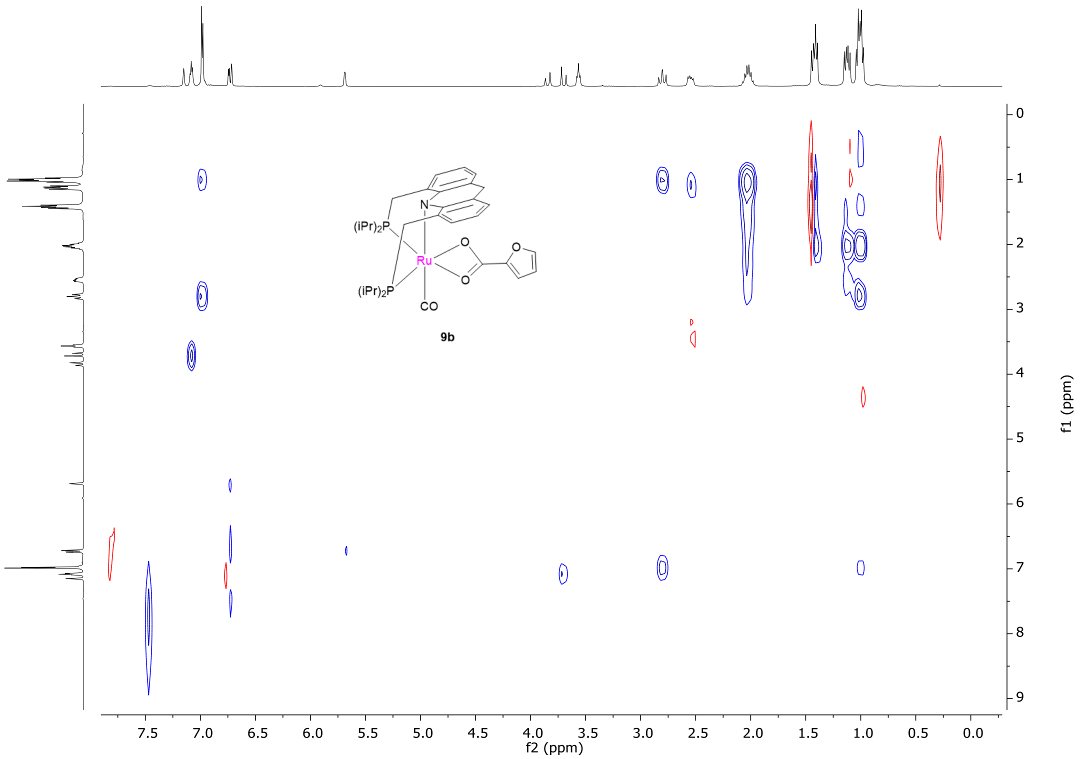

Figure S27. ^{1}H-^{1}H NOSEY NMR spectrum of complex **9b** in C_6D_6.

6.2. $^{31}P\{^1H\}$ and ^1H NMR spectra of complex 6 in C_6D_6

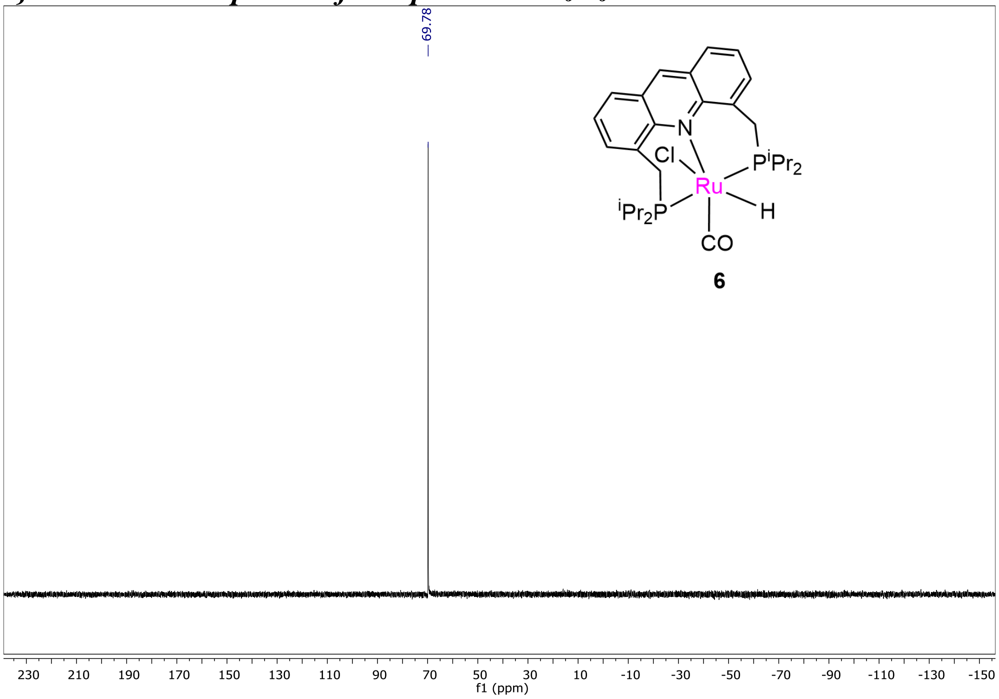

Figure S28. $^{31}P\{^1H\}$ NMR (162 MHz) of complex **6** in C_6D_6. Chemical shifts are in accordance with previously reported.[6]

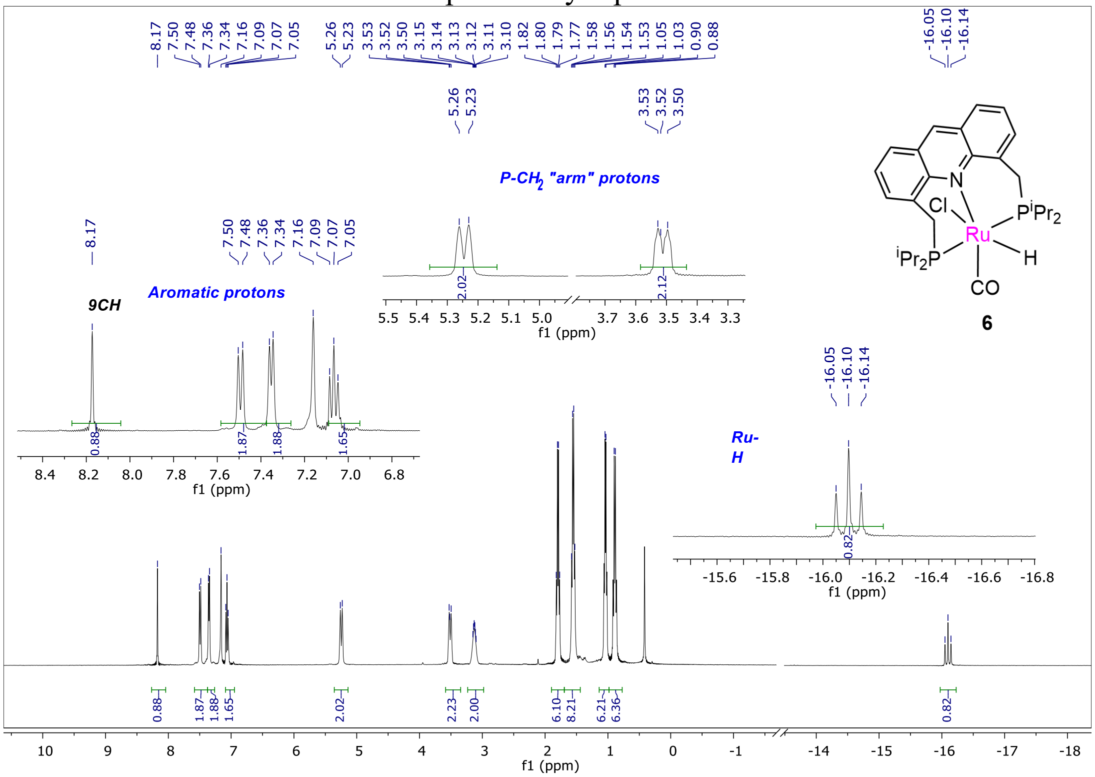

Figure S29. ^1H NMR (400 MHz) of complex **6** in C_6D_6. Chemical shifts are in accordance with previously reported.[6]

6.3. ^{1}H and $^{31}P\{^{1}H\}$ NMR spectra of dearomatized complex 9

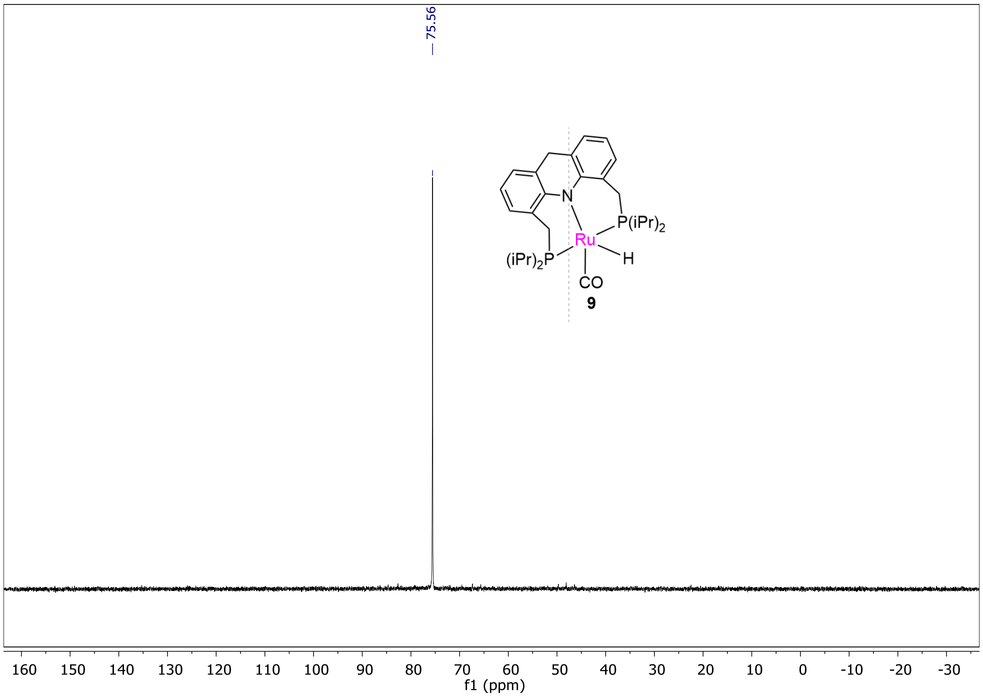

Figure S30. $^{31}P\{^{1}H\}$ NMR spectrum (162 MHz) of complex **9** in C_6D_6. Chemical shifts are in accordance with previously reported.[8]

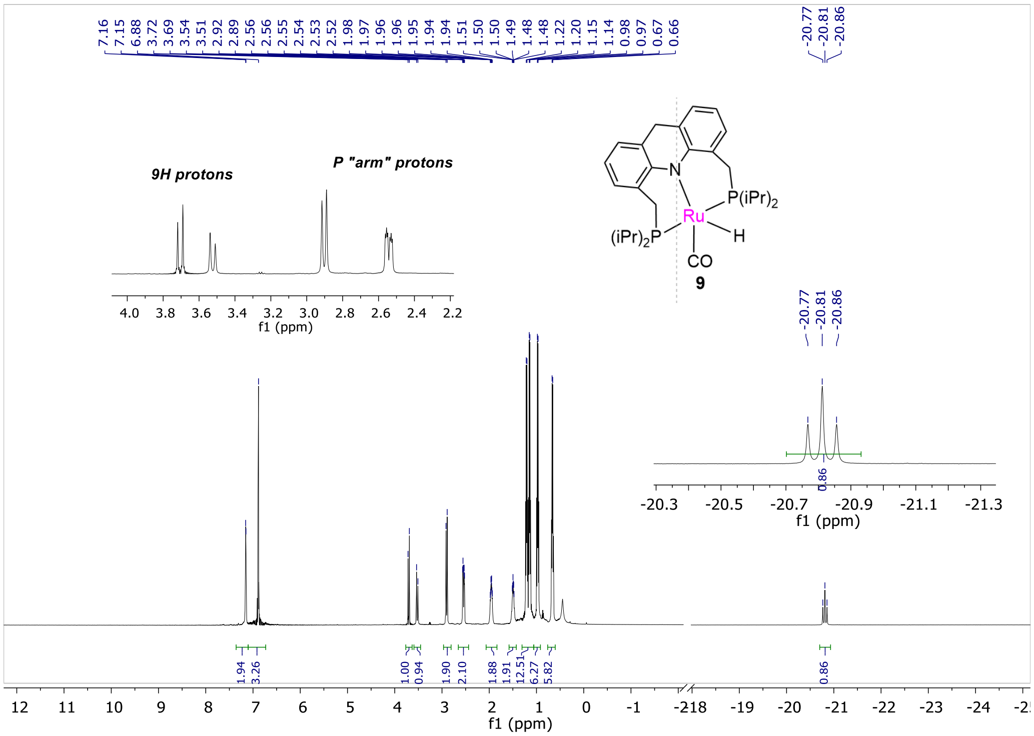

Figure S31. ^{1}H NMR spectrum (400 MHz) of complex **9** in C_6D_6. Chemical shifts are in accordance with previously reported. [8]

6.4. Reactivity of complexes as followed by ^{1}H and ^{31}P{^{1}H} NMR studies

6.4.1. *Complex **6**, in the presence of benzaldehyde, NaOH and water, forms the dearomatized complex **9** and dearomatized benzoate complex **9a** upon heating*

Procedure. In a J Young NMR tube was added a mixture of 5 μmol of complex **6**, 0.025 mmol of benzaldehyde and 0.1 mL water in 0.5 mL of THF. The tube was then heated at 130 °C for 10 min and the ^{31}P{^{1}H} and ^{1}H NMR were subsequently recorded. No formation of any new complex was observed in the NMR. Subsequently, the tube was taken inside glove box and 0.05 mmol of NaOH was added. The mixture was then heated again at 130 °C for 10 min and the NMR spectra was measured, which revealed the formation of two distinct complexes. The complexes were assigned to the dearomatized acridine hydride complex **9**[12] and the dearomatized acridine benzoate complex **9a**,[8] based on the chemical shift analysis compared to the previously reported data in the literature.

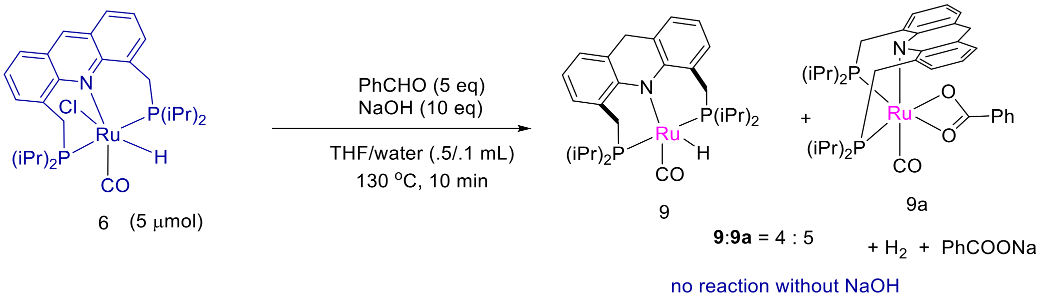

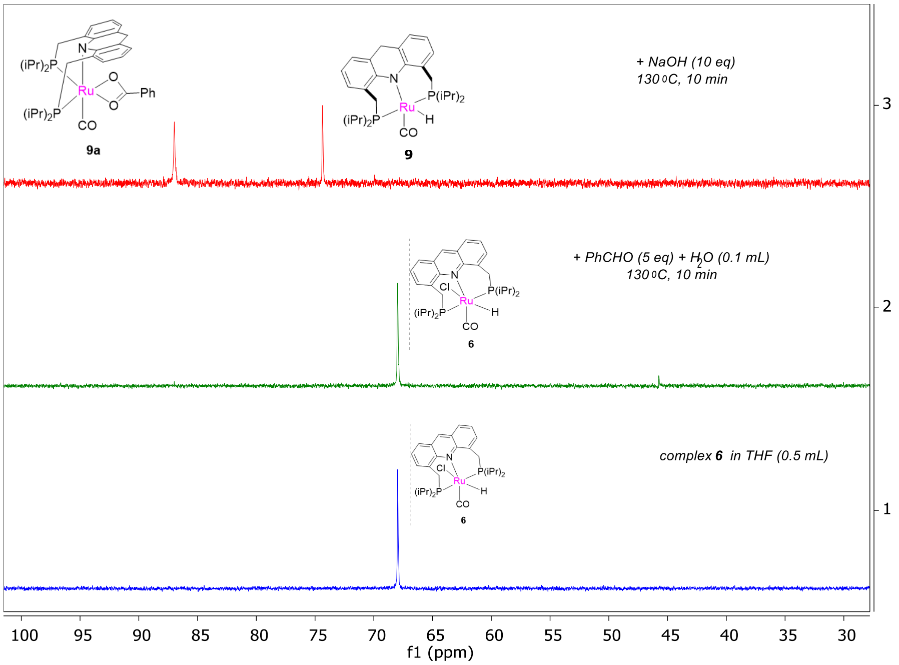

Figure S32. Stacked ^{31}P{^{1}H} NMR spectra of reaction between complex **6**, benzaldehyde, NaOH and water.

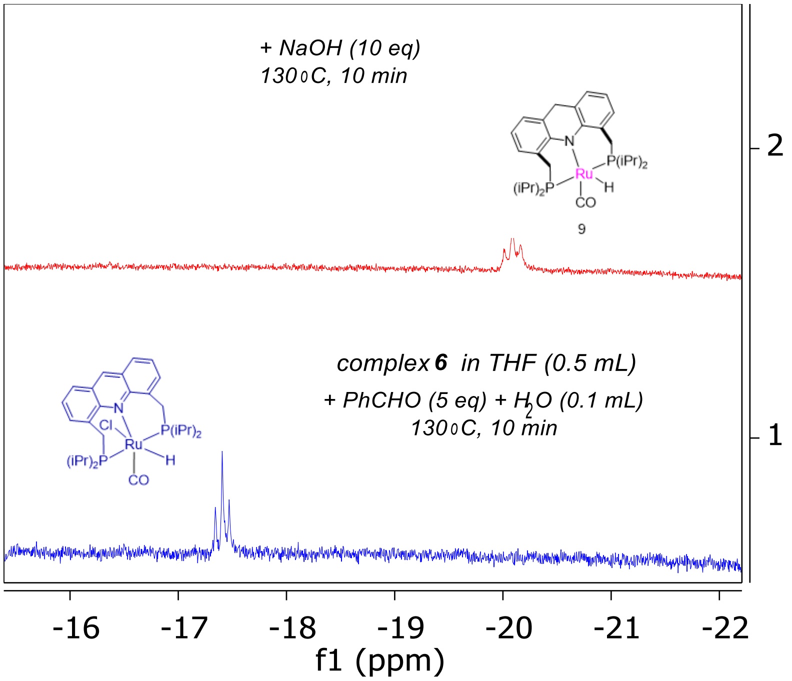

Figure S33. Stacked $^{31}P\{^{1}H\}$ NMR spectra of reaction between complex **6**, benzaldehyde, NaOH and water.

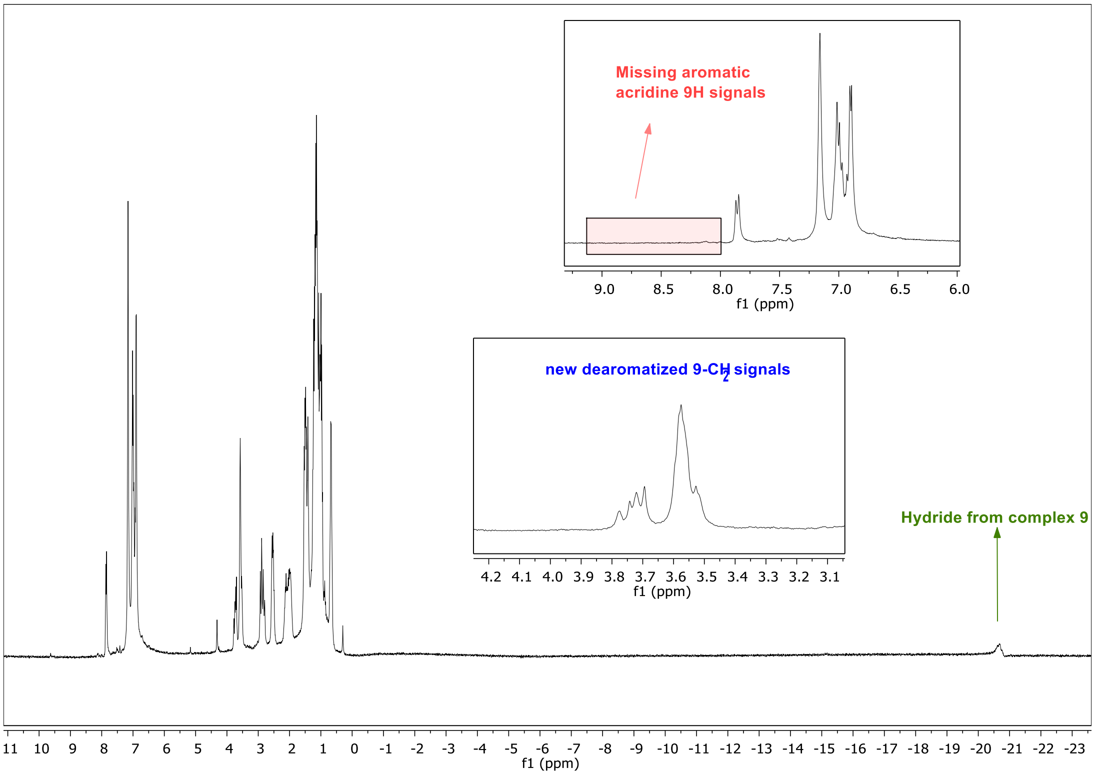

Figure S34. ^{1}H NMR of the complexes after reaction after extraction in C_6D_6. Insets show the disappearance of 9H aromatic signals and appearance of new $9CH_2$ signals.

*6.4.2. Complex **9**, in the presence of benzaldehyde, and water, forms the dearomatized benzoate complex **9a** at RT*

Procedure. To a J Young NMR tube was added a solution of 5 µmol of complex **9** in 0.5 mL THF. To the solution, 0.025 mmol of benzaldehyde and 0.05 mmol water was added. The tube was then stirred at room temperature for 10 min and the ^{31}P and ^{1}H NMR was subsequently recorded. Formation of the dearomatized benzoate complex **9a** was observed by NMR spectroscopy.

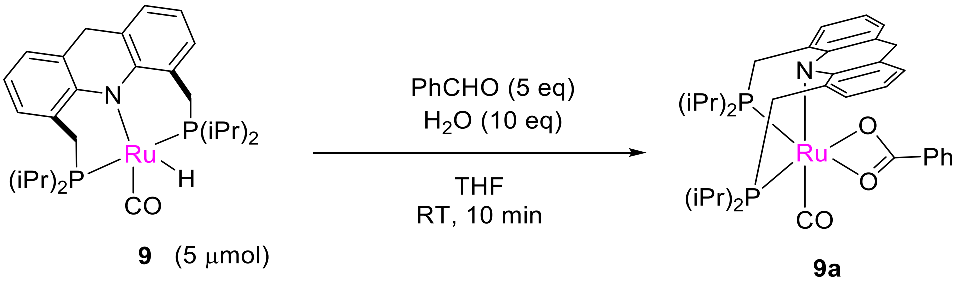

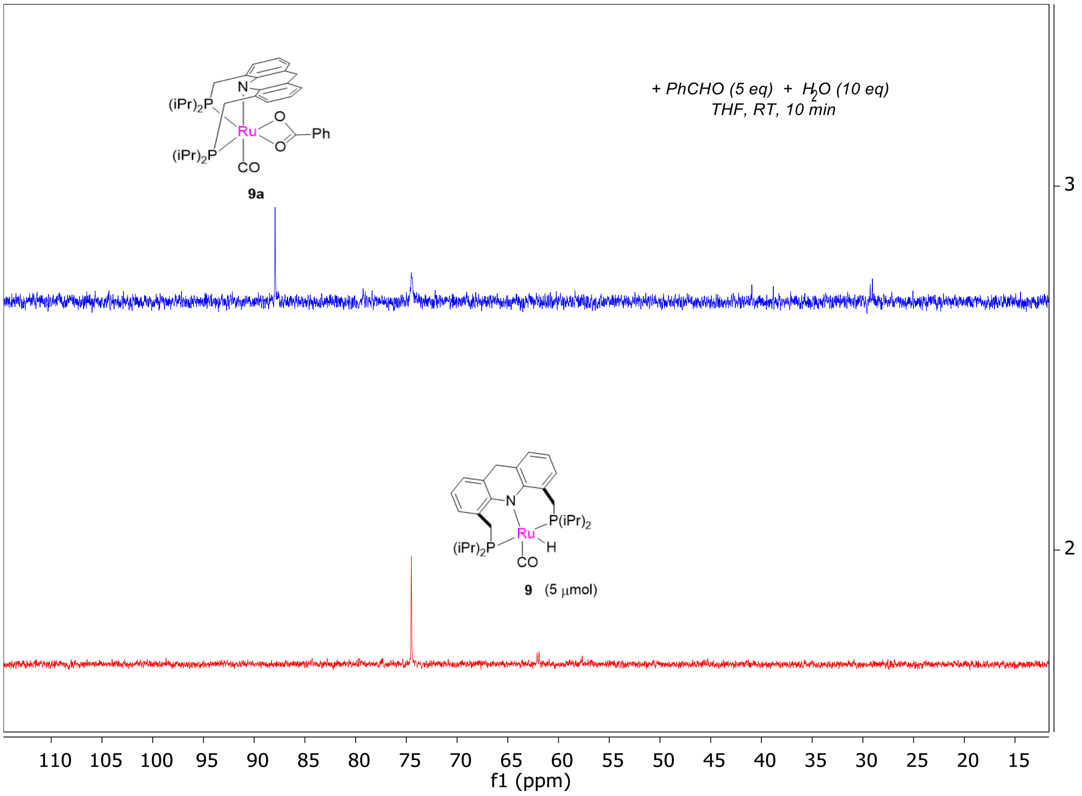

Figure S35. Stacked $^{31}P\{^{1}H\}$ NMR spectra of reaction between complex **9**, benzaldehyde, and water.

*6.4.3. Complex **9**, in the presence of furfural and water, forms the dearomatized furoate complex **9b** upon heating*

Procedure. To a J Young NMR tube was added a solution of 5 μmol of complex **9** in 0.5 mL THF. To the solution, 0.025 mmol of furfural and 0.1 mL water was added. The tube was then stirred at room temperature for 10 min and the ^{31}P and ^{1}H NMR spectra were subsequently recorded. Formation of several unidentifiable species was observed in the ^{31}P NMR spectrum. The solution was then heated at 130 ^{o}C for 10 min and the NMR spectra were measured again, revealing the formation the furoate complex **9b** as the major species.

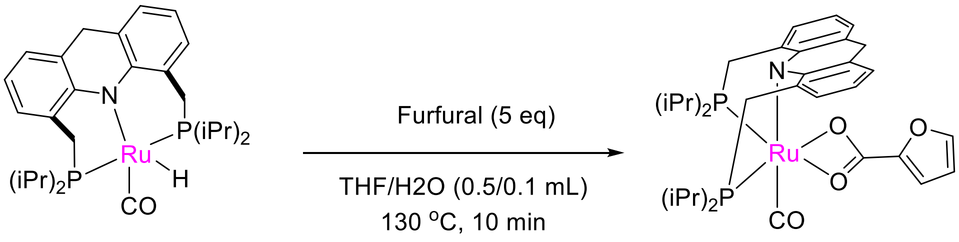

9 (5 μmol)

9b

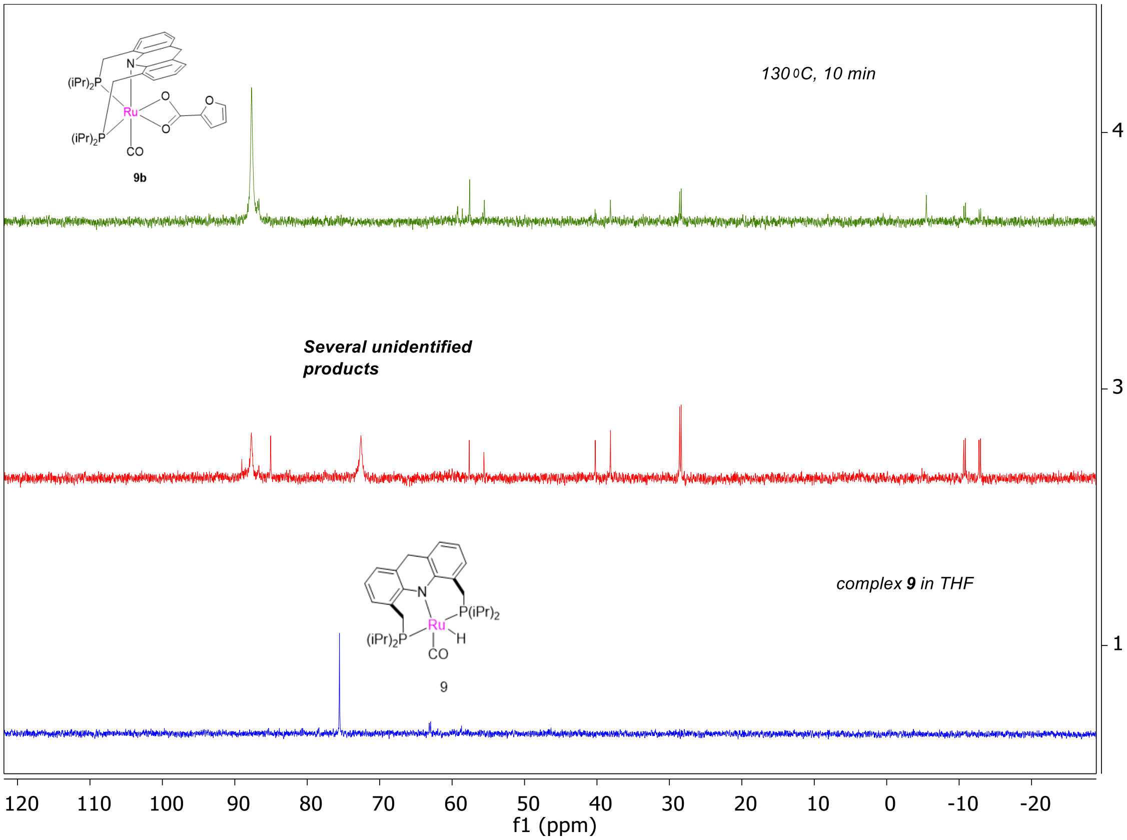

Figure S36. Stacked ^{31}P{^{1}H} NMR spectra of reaction between complex **9**, furfural, and water.

*6.4.4. The furoate complex **9b** can also be accessed from **9** by reaction with furoic acid*

Procedure. In a J Young NMR tube was added a solution of 0.01 mmol of complex **9** in 0.5 mL of THF, to which 0.012 mmol of furoic acid was added. The tube was then stirred at room temperature for 10 min and the ^{31}P and ^{1}H NMR were subsequently recorded. Formation of the furoate complex **9b** was observed as the major species along with the *mer* furoic acid adduct complex (**9e**) as the minor species. The NMR was again taken after 1 h of stirring and the furoate complex **9b** was observed as the only species.

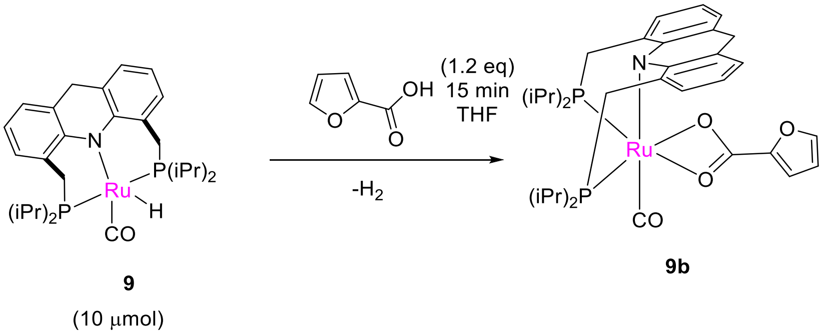

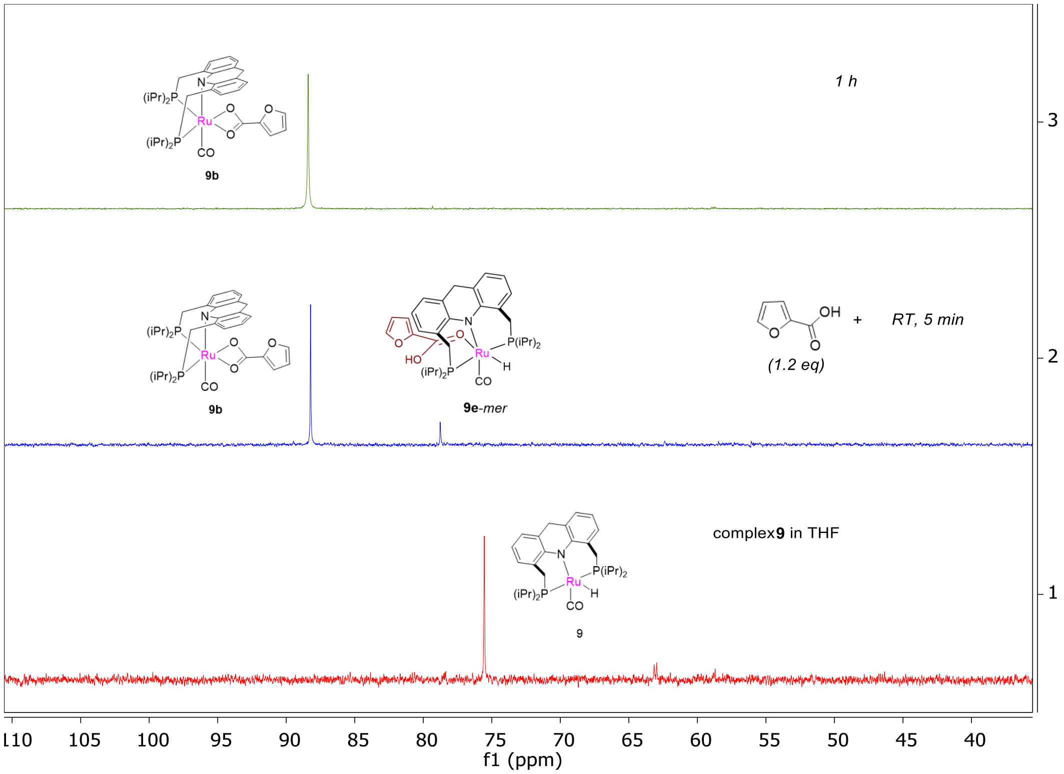

Figure S37. Stacked $^{31}P\{^{1}H\}$ NMR spectra of reaction between complex **9**, and furoic acid.

*6.4.5. The furoate complex **9b** is stable in the presence of water in THF even at elevated temperatures*

Procedure. The furoate complex was prepared *in situ* by mixing **9** with furoic acid in THF. To a J Young tube, 0.5 mL THF solution of 5 μmol complex **9b** was added. 0.05 mmol of water was then added and the resulting solution was stirred at room temperature for 10 min and the ^{31}P and ^{1}H NMR were subsequently recorded. No reaction was observed by ^{31}P NMR with only the furoate complex **9b** being present in solution. Subsequently, the tube was heated at 130 °C for 10 min and the NMR spectra were recorded again. The furoate complex **9b** was observed as the only species.

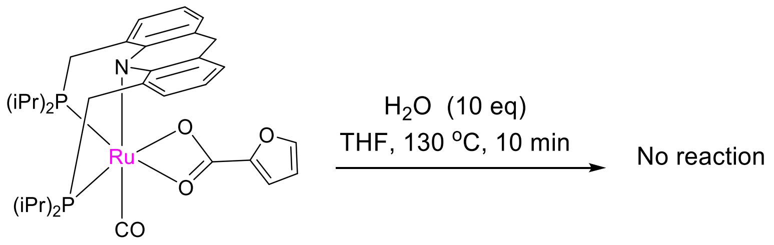

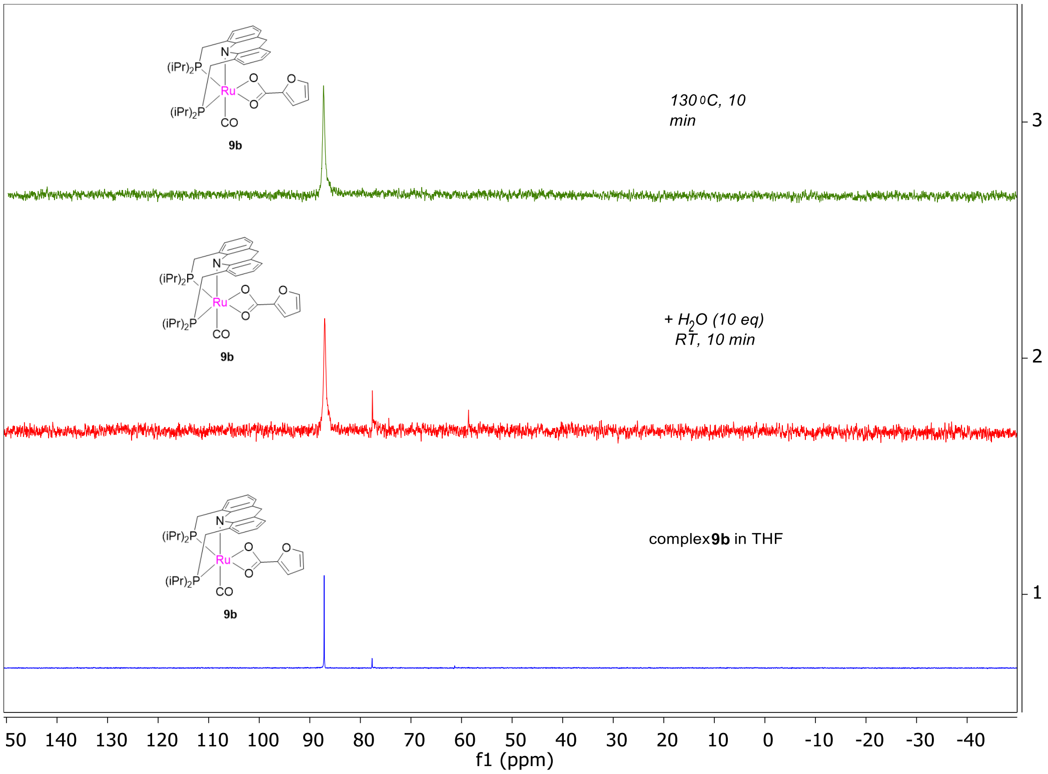

Figure S38. Stacked $^{31}P\{^{1}H\}$ NMR spectra of reaction between complex **9b** and water.

*6.4.6. The furoate complex **9b** forms a new complex upon addition of base, which decomposes when heated to high temperature*

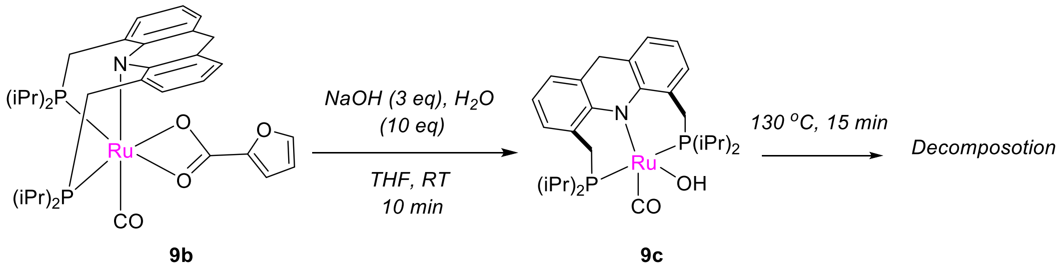

Procedure. To the NMR tube from previous experiment, NaOH (0.015 mmol) was added. The resulting solution was stirred for 10 min and the ^{31}P and ^{1}H NMR were subsequently recorded. Formation of a new species, likely the ruthenium hydroxide complex was observed, along with generation of sodium furoate in the ^{1}H NMR. Subsequently, the tube was heated at 140 ºC for 10 min and the NMR were recorded again. Decomposition of the previous complex to multiple unidentified species was observed. Complex **9c** is also observed to decompose slowly at RT to other complexes or upon isolation attempts by extraction in benzene, likely signifying the requirement of alkaline conditions for its prolonged stability.

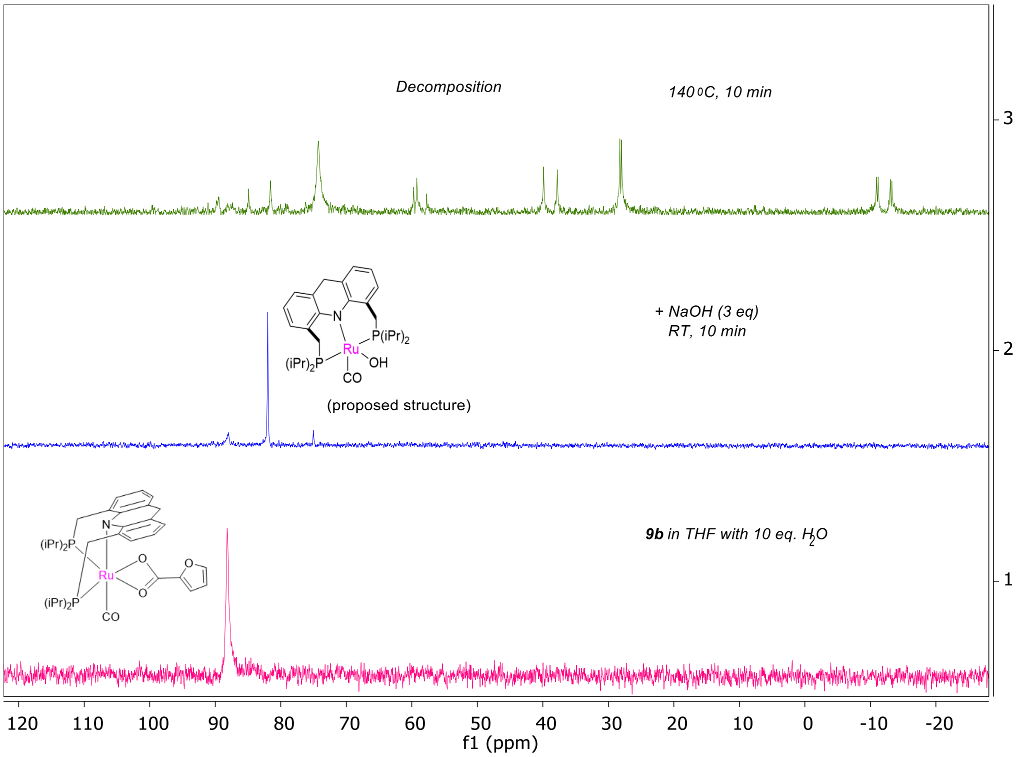

Figure S39. Stacked $^{31}P\{^{1}H\}$ NMR spectra of reaction between complex **9b**, water and NaOH.

7. General discussion

7.1. Possible catalytic cycles for alcohol dehydrogenation

The possible catalytic cycles for furfuryl alcohol dehydrogenation to furfural are shown below. Under the reaction conditions, two alternative catalytic cycles are possible. In the water unassisted pathway (in blue), initial coordination of furfuryl alcohol, followed by H_2 evolution can generate the alkoxy complex **9f**. Further β hydride elimination from the alkoxy complex can then generate complex **9**-*fac* and furfural. Previous computational studies regarding ethanol dehydrogenation by complex **9** had suggested that the initial H_2 evolution step is rate determining step.[13] In the presence of water, an alternative stepwise water assisted dehydrogenation pathway can also be envisioned (in red).[9] In this mechanism, initial water coordination followed by dehydrogenation generates the hydroxy intermediate **9c**. Further alcohol coordination to **9c** followed by water elimination generates alkoxide intermediate **9f**, in this alternative water assisted stepwise pathway. The generated aldehyde, after subsequent β hydride elimination, converts to furoate salt via dehydrogenative oxidation or disproportionation pathway, as discussed in the main text.

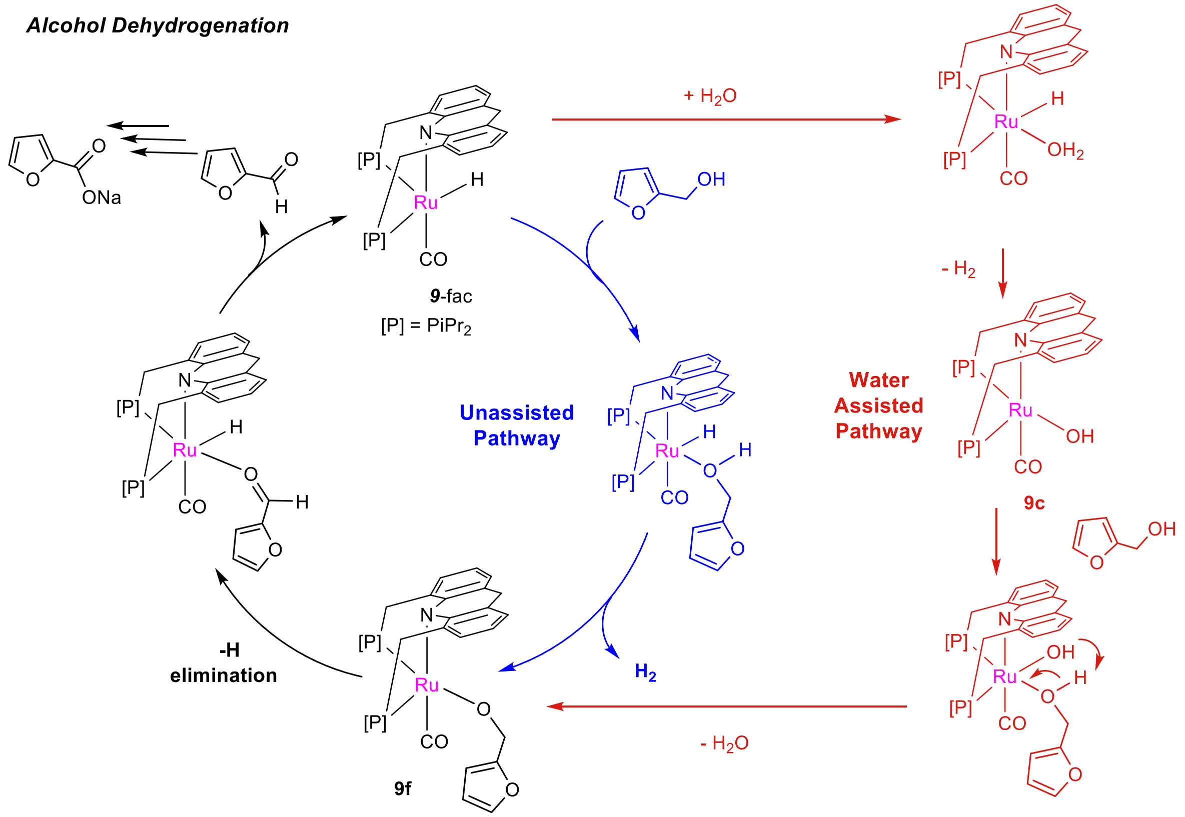

Figure S40. Possible catalytic pathways for alcohol dehydrogenation catalyzed by complex **9**.

7.2. Possible catalytic cycle for Tishchenko coupling

Complex **9** was observed to catalyze the Tishchenko coupling of furfurals to furfuryl furoate ester in 1,4-dioxane (Figure S19). The tentative mechanism cycle of the process is given below. Initial coordination of aldehyde to **9** followed by hydride transfer generates the alkoxy complex **9f**. To **9f**, another furfural coordinates in the *cis* vacant site. Furfuryloxy anion migration to bound furfural can further generate the acetal complex **9g**. Finally, β hydride elimination takes place from the acetal complex to generate the ester and complex **9**-*fac*. A relevant computational study for ester formation from aldehydes by complex **9** has been reported recently by our group.[13]

Tentative mechanism for Tishchenko Coupling

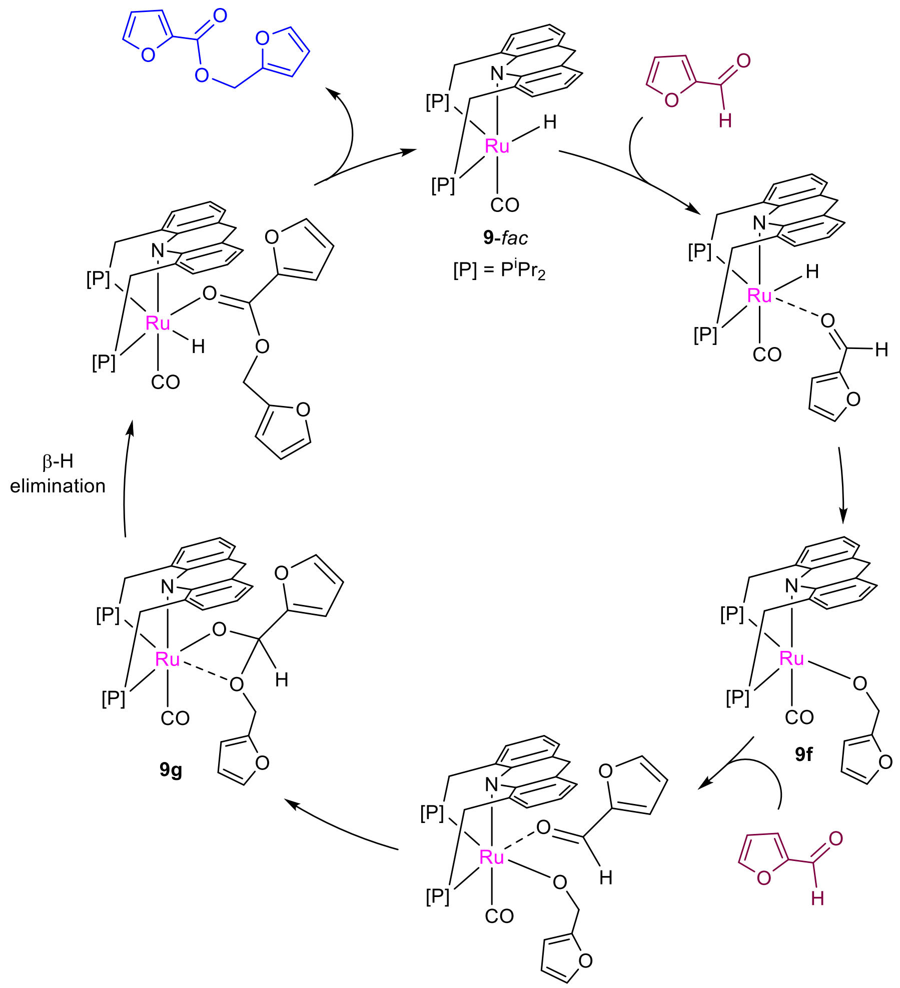

Figure S41. Possible catalytic cycle for furfural disproportionative coupling to ester catalyzed by complex **9.**

7.3. Different ways to access the dearomatized complex 9 from the aromatized complex 6

The dearomatized complex **9** can be accessed from the aromatized complex **6** via several different hydride transfer methods—

1) By heating a solution of **6** in H_2 with a base KOH.[6]
2) By the treatment of **6** with super-hydride $NaBEt_3H$ at room temperature.[8]
3) By heating a solution of **6** with alcohol/aldehyde substrate in presence of base.[7,9] In this last case, one C-H proton is transferred to the 9 position of the catalyst acridine ring to generate dearomatized complex while oxidizing the hydride donor (aldehyde from alcohol; acid from aldehyde)

These different routes are shown in the figure below. However, it should be noted that the dearomatization of the acridine ring is seemingly irreversible, and we have not yet observed the formation of aromatic complexes from the dearomatized complexes under the reaction conditions in this study or the previous studies.

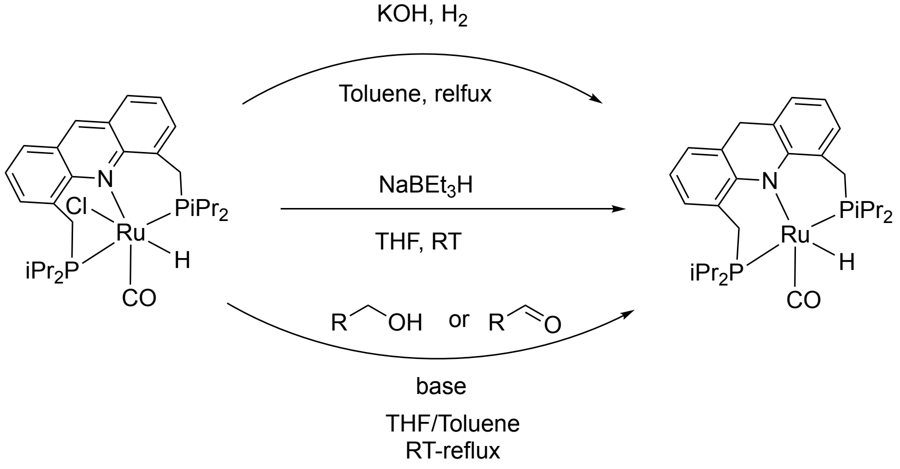

Figure S42. Different ways to access the dearomatized complex **9** from **6**.

7.4. Oxidation of other aldehydes using alkaline water catalyzed by 6

We also explored the possibility of oxidizing other aldehydes by the developed protocol herein. Under the reaction conditions (similar procedure as in section 3), benzaldehyde, hexanal, and phenylacetaldehyde can also be converted to the corresponding acids in near quantitative yields, demonstrating the versatility of the process.

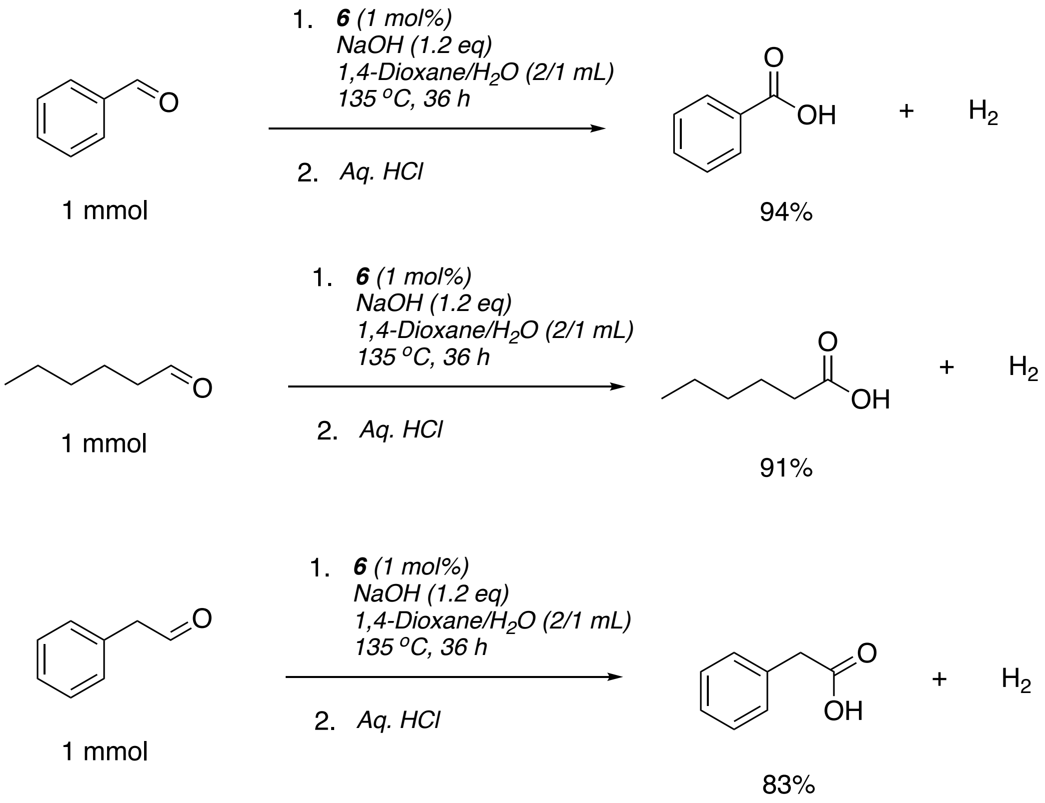

Figure S43. Oxidation of other aldehydes by alkaline water catalyzed by **6**

7.5. Alternate reaction mechanism involving free acetal dehydrogenation

An alternate mechanistic cycle involving the direct water attack on the aldehyde to form free acetal, followed by acetal dehydrogenation can also be envisioned. In this mechanism, the free acetal can coordinate to complex **9** to generate the acetal adduct complex **9h**. H_2 extrusion from **9h** will then generate the chelating acetal complex **9d**. Subsequently, beta hydride elimination from **9d** will generate the acid adduct complex **9f**. Further, **9f**.can generate the product acid salt and complex **9** in the presence of a base. In this mechanism, the carboxylate complex **9b** is not a part of the mechanistic cycle but acts as an off cycle resting intermediate.

Based on the experimental observations, we cannot entirely rule out this alternate mechanism. Although, given the easy formation of carboxylate complex **9b** from **9f**, and further equilibrium of the hydroxy complex (**9c**) with the carboxylate complex (**9b**) that is observed in presence of NaOH, the mechanism as proposed in the manuscript seems more in line with the experiments. It will be interesting to explore these competing mechanisms by DFT studies in future to find the most energetically favorable reaction pathway.

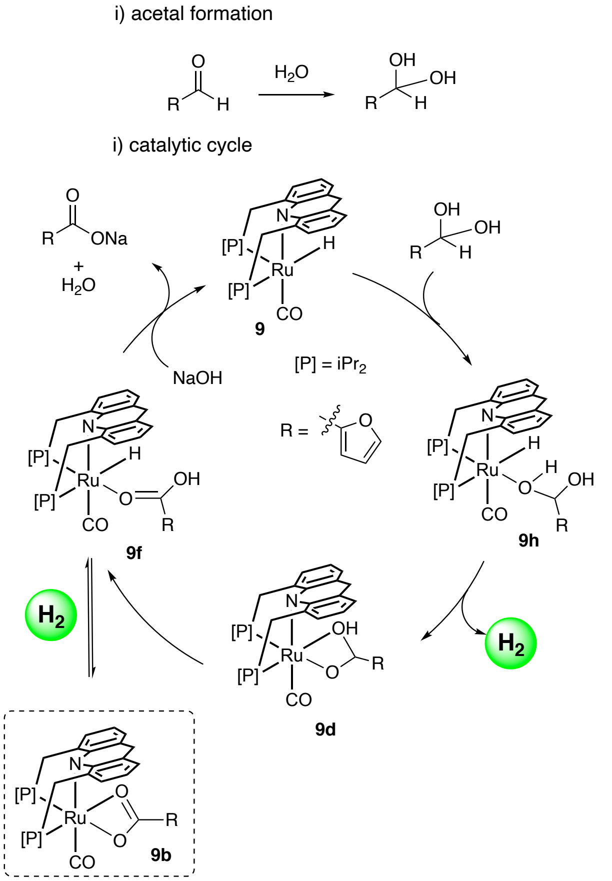

Figure S44. Alternate mechanistic cycle based on free acetal dehydrogenation

7.6. Summary of the overall proposed pathways

A summary of the overall ongoing pathways is shown in Figure S45. The five fundamental reactions ongoing under the conditions are— a) [Ru] catalyzed aldehyde oxidation by water to acid salt, b) base catalyzed Cannizzaro disproportionation of the aldehyde to acid and alcohol, c) [Ru] catalyzed Tishchenko coupling of aldehyde to ester, d) base mediated ester hydrolysis to acid and alcohol and e) [Ru] catalyzed alcohol dehydrogenation to the aldehyde. For furfural, all these pathways eventually lead to the formation of furoic acid salt in quantitative yield, whose formation is also irreversible. For HMF oxidation to FDCA, initially the aldehyde group is oxidized to acid fully via the combination of these pathways, and at the next step, the alcohol group is oxidized to acid (which is more challenging to oxidize than the aldehyde group under the reaction conditions), generating the desired product FDCA.

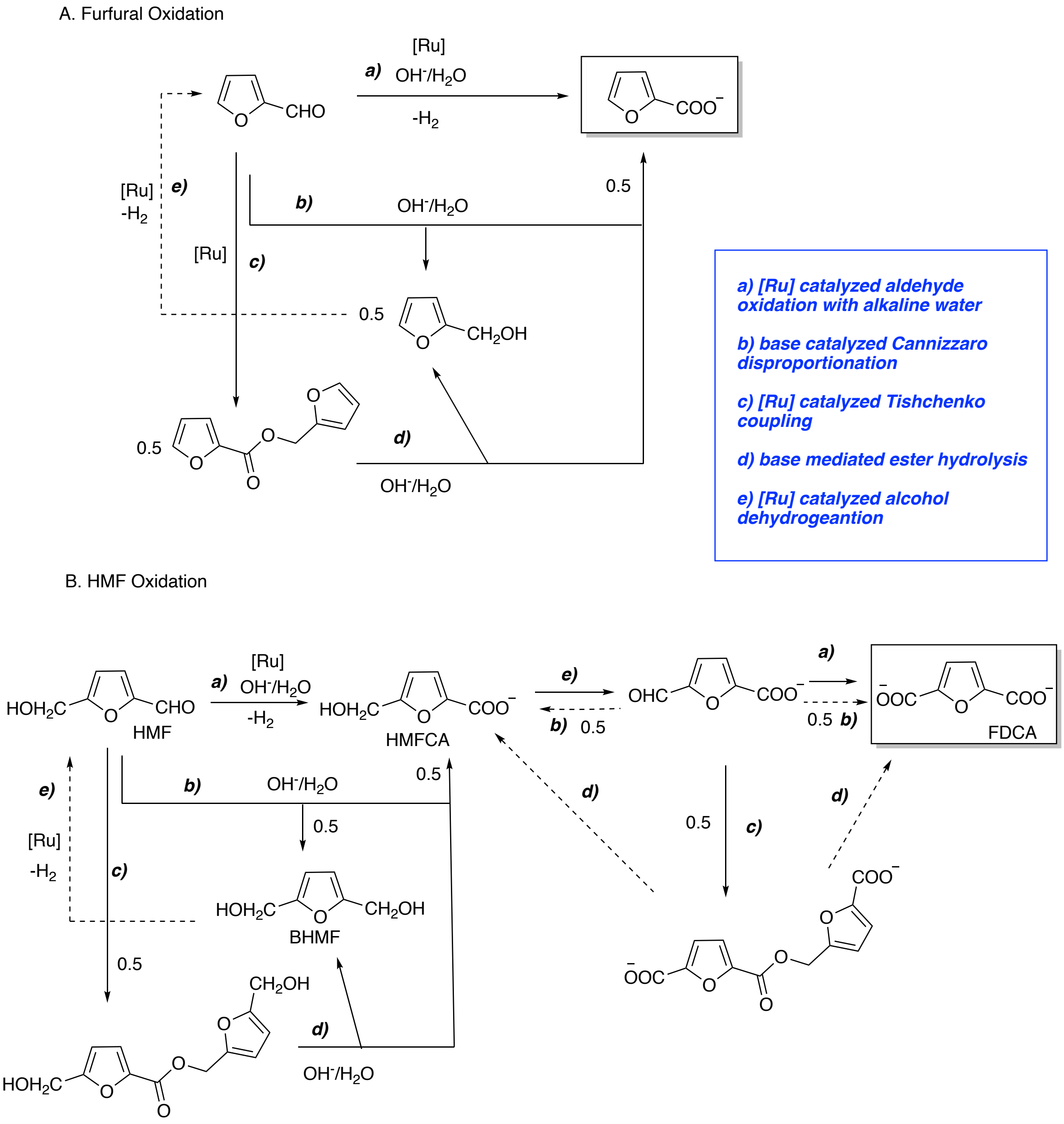

Figure S45. Summary of the ongoing pathways during furfural (A) and HMF (B) oxidation.

8. References

1. Balaraman, E.; Gnanaprakasam, B.; Shimon, L. J. W.; Milstein, D., Direct Hydrogenation of Amides to Alcohols and Amines under Mild Conditions. *J. Am. Chem. Soc.* **2010,** *132*, 16756-16758.
2. Gunanathan, C.; Ben-David, Y.; Milstein, D., Direct Synthesis of Amides from Alcohols and Amines with Liberation of H_2. *Science* **2007,** *317*, 790.
3. Gnanaprakasam, B.; Zhang, J.; Milstein, D., Direct Synthesis of Imines from Alcohols and Amines with Liberation of H2. *Angew. Chem. Int. Ed.* **2010,** *49*, 1468-1471.
4. Fogler, E.; Garg, J. A.; Hu, P.; Leitus, G.; Shimon, L. J. W.; Milstein, D., System with Potential Dual Modes of Metal–Ligand Cooperation: Highly Catalytically Active Pyridine-Based PNNH–Ru Pincer Complexes. *Chem. - Eur. J.* **2014,** *20*, 15727-15731.
5. Srimani, D.; Balaraman, E.; Hu, P.; Ben-David, Y.; Milstein, D., Formation of Tertiary Amides and Dihydrogen by Dehydrogenative Coupling of Primary Alcohols with Secondary Amines Catalyzed by Ruthenium Bipyridine-Based Pincer Complexes. *Adv. Synth. Catal.* **2013,** *355*, 2525-2530.
6. Gunanathan, C.; Gnanaprakasam, B.; Iron, M. A.; Shimon, L. J. W.; Milstein, D., "Long-Range" Metal−Ligand Cooperation in H_2 Activation and Ammonia-Promoted Hydride Transfer with a Ruthenium−Acridine Pincer Complex. *J. Am. Chem. Soc.* **2010,** *132*, 14763-14765.
7. Ye, X.; Plessow, P. N.; Brinks, M. K.; Schelwies, M.; Schaub, T.; Rominger, F.; Paciello, R.; Limbach, M.; Hofmann, P., Alcohol Amination with Ammonia Catalyzed by an Acridine-Based Ruthenium Pincer Complex: A Mechanistic Study. *J. Am. Chem. Soc.* **2014,** *136*, 5923-5929.
8. Tang, S.; Rauch, M.; Montag, M.; Diskin-Posner, Y.; Ben-David, Y.; Milstein, D., Catalytic Oxidative Deamination by Water with H2 Liberation. *J. Am. Chem. Soc.* **2020,** *142*, 20875-20882.
9. Gellrich, U.; Khusnutdinova, J. R.; Leitus, G. M.; Milstein, D., Mechanistic Investigations of the Catalytic Formation of Lactams from Amines and Water with Liberation of H_2. *J. Am. Chem. Soc.* **2015,** *137*, 4851-4859.
10. Zhang, X.; Zhang, W.-Z.; Shi, L.-L.; Guo, C.-X.; Zhang, L.-L.; Lu, X.-B., Silver(i)-catalyzed carboxylation of arylboronic esters with CO2. *Chem. Commun.* **2012,** *48*, 6292-6294.
11. Yi, G.; Teong, S. P.; Zhang, Y., Base-free conversion of 5-hydroxymethylfurfural to 2,5-furandicarboxylic acid over a Ru/C catalyst. *Green Chem.* **2016,** *18*, 979-983.
12. Zou, Y.-Q.; von Wolff, N.; Anaby, A.; Xie, Y.; Milstein, D., Ethylene glycol as an efficient and reversible liquid-organic hydrogen carrier. *Nat. Catal.* **2019,** *2*, 415-422.
13. Rauch, M.; Luo, J.; Avram, L.; Ben-David, Y.; Milstein, D., Mechanistic Investigations of Ruthenium Catalyzed Dehydrogenative Thioester Synthesis and Thioester Hydrogenation. *ACS Catal.* **2021**, *11,* 2795-2807.
